# Inversion polymorphism in a complete human genome assembly

**DOI:** 10.1186/s13059-023-02919-8

**Published:** 2023-04-30

**Authors:** David Porubsky, William T. Harvey, Allison N. Rozanski, Jana Ebler, Wolfram Höps, Hufsah Ashraf, Patrick Hasenfeld, Human Pangenome Reference Consortium (HPRC), Human Genome Structural Variation Consortium (HGSVC), Benedict Paten, Ashley D. Sanders, Tobias Marschall, Jan O. Korbel, Evan E. Eichler

**Affiliations:** 1 Department of Genome Sciences, University of Washington School of Medicine, Seattle, WA 98195 USA; 2 Institute for Medical Biometry and Bioinformatics, Medical Faculty, Heinrich Heine University, Moorenstraße 5, 40225 Düsseldorf, Germany; 3 European Molecular Biology Laboratory (EMBL), Genome Biology Unit, Meyerhofstr. 1, 69117 Heidelberg, Germany; 4 UC Santa Cruz Genomics Institute, University of California Santa Cruz, Santa Cruz, CA 95064 USA; 5 Berlin Institute for Medical Systems Biology, Max Delbrück Center for Molecular Medicine, Helmholtz Association, 10115 Berlin, Germany; 6 Berlin Institute of Health (BIH), 10178 Berlin, Germany; 7 Charité-Universitätsmedizin, 10117 Berlin, Germany; 8 Center for Digital Medicine, Heinrich Heine University, Moorenstraße 5, 40225 Düsseldorf, Germany; 9 European Molecular Biology Laboratory, European Bioinformatics Institute, Wellcome Genome Campus, Hinxton, Cambridge, CB10 1SD UK; 10 Howard Hughes Medical Institute, University of Washington, Seattle, WA 98195 USA

**Keywords:** Genomic structural variation, Inversion, Pathogenic copy number variant, T2T-CHM13, Pericentromeric

## Abstract

The telomere-to-telomere (T2T) complete human reference has significantly improved our ability to characterize genome structural variation. To understand its impact on inversion polymorphisms, we remapped data from 41 genomes against the T2T reference genome and compared it to the GRCh38 reference. We find a ~ 21% increase in sensitivity improving mapping of 63 inversions on the T2T reference. We identify 26 misorientations within GRCh38 and show that the T2T reference is three times more likely to represent the correct orientation of the major human allele. Analysis of 10 additional samples reveals novel rare inversions at chromosomes 15q25.2, 16p11.2, 16q22.1–23.1, and 22q11.21.

**Supplementary Information:**

The online version contains supplementary material available at 10.1186/s13059-023-02919-8.

## Background

A gapless telomere-to-telomere (T2T) assembly of a human genome (T2T-CHM13) was recently released [1]. The complete reference newly resolved > 240 Mbp of sequence not previously represented in GRCh38 improving the discovery of single-nucleotide variants [2] and copy number variants (CNVs) [3]. Compared to other classes of variation, the detection of balanced events such as inversions is particularly challenging [4]. This is because most inversions are copy number neutral and are associated with repetitive DNA [5–7]. This is especially true for the largest events that are frequently flanked by long and highly identical segmental duplications (SDs). Even among existing high-quality long-read genome assemblies, large inversion polymorphisms are often missed or incorrectly represented [8]. While various approaches have been developed over the years to detect inversions (including mate-pair detection, optical mapping, Strand-seq, and long-read sequence detection), a combination of these methods has been shown to produce the best results. Long-read sequencing methods are particularly powerful for detecting smaller inversions (< 10 kbp) that occur in relatively repeat-free regions of the genome. In contrast, the Strand-seq platform remains among the most sensitive for detection of large inversions (> 10 kbp) that are flanked by SDs [7, 9] and affect the greatest number of base pairs per haploid genome. Accurate detection of inversions of this type is critical for understanding human variation and disease because recurrent inversions have been shown to associate with regions of genome instability and neurodevelopmental disease [10–16].

The T2T-CHM13 assembly has been put forward as an improved human reference genome over the current incomplete GRCh38 and GRCh37 references. We sought to assess the potential advantage of detecting inversions on this new reference when compared to GRCh38 and whether it would significantly alter our understanding of the landscape and frequency of inversion polymorphism in the human genome. We, therefore, recalled inversions with respect to the T2T-CHM13 reference using data from multiple genomic platforms (Strand-seq, Bionano, and long-read assemblies) for 41 human genomes of diverse population origin [7].

## Results and discussion

### More accurate and complete inversion discovery with T2T reference

**Fig. 1:**
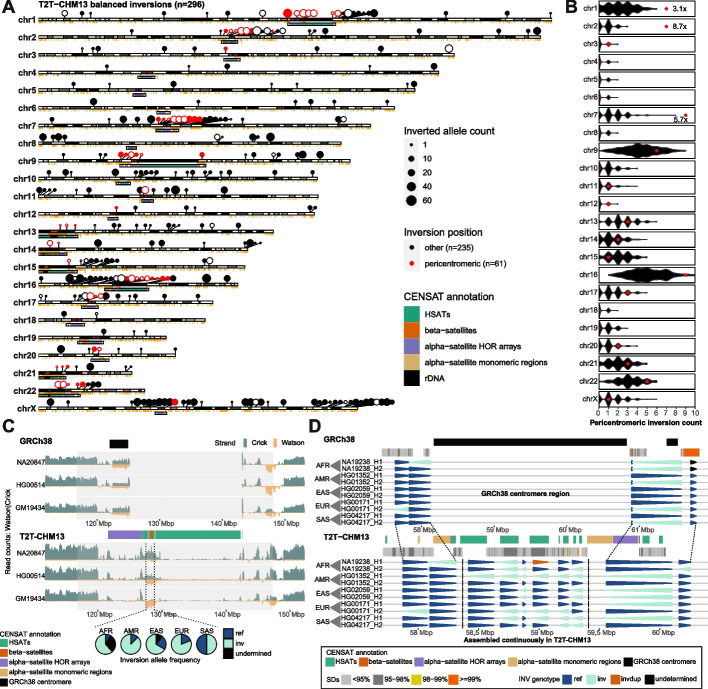
Inversion polymorphisms with respect to a complete T2T reference show pericentromeric bias. **A** An ideogram showing the position and inverted allele frequency (dot size) of all balanced inversions from 41 human samples mapped to T2T-CHM13 reference (*n* = 296). Inversions that fall within pericentromeric regions (CENSAT annotation, ± 1 Mbp) are shown as red dots (*n* = 61) while other inversions are shown as black dots (*n* = 235). Inversions with ≥ 90% reciprocal overlap with nonsyntenic regions between GRCh38 and T2T-CHM13 or that failed to map to the GRCh38 reference are highlighted as open circles (*n* = 63). **B** Permutation analysis shows pericentromeric enrichment for specific chromosomes. Permuted counts of pericentromeric inversions are shown as black violin plots as compared to observed counts (red dots). **C** The read-coverage profiles of Strand-seq data over a chromosome 1 centromeric region summarized as binned (bin size: 50 kbp step size: 10 kbp) read counts represented as bars above (teal; Crick read counts) and below (orange; Watson read counts) the midline with respect to centromere repeat annotation. Dotted lines highlight the novel centromeric inversion detected on chromosome 1 only with respect to T2T-CHM13. Note: equal coverage of Watson and Crick counts represent a heterozygous inversion (one homologue inverted) while reads aligned only in the Watson orientation signify a homozygous inversion (both homologs inverted). Pie charts show frequency of inverted (bright blue) and directly oriented (light blue) alleles across all haplotypes (*n* = 82) from all unrelated individuals (*n* = 41) for a given centromeric inversion (dotted lines). **D** A “backgammon” plot showing the inversion status of each defined region reported as colored arrowheads (dark blue—direct, bright blue—inverted, see the legend) for chromosome 7 region with respect to GRCh38 (chr7:57456486–61949954; top) and T2T-CHM13 (chr7:57700000–60400000; bottom). *HSATs* human satellites, *HOR *higher-order repeat

Previously, we generated Strand-seq data from 41 samples from the 1000 Genomes Project. Using the same algorithm applied to GRCh38, we remapped the Strand-seq data to T2T-CHM13 (v1.1) and combined it with both Bionano Genomics and assembly-based approaches to detect inversions [7] (Methods). With this reanalysis we identified 373 inverted regions, including 296 balanced inversions, 56 inverted duplications, and 21 complex events across the autosomes and chromosome X (Additional files 1: Fig. S1A and Additional file 2: Table S1). For the remainder of this study, we focus exclusively on analysis of 296 balanced inversions (referred to as inversions or inversion polymorphisms), because they can be accurately and comprehensively genotyped to establish meaningful population frequencies (Fig. 1A, Additional file 1: Supplemental Notes). While we report a comparable number of inverted bases per chromosome (Additional file 1: Fig. S1B), the T2T-CHM13 callset increases the total number of inverted bases by ~ 10.5 Mbp (82.8 Mbp) compared to GRCh38 (72.3 Mbp). In addition, the GRCh38 reference harbors 26 misorientations—defined here as any region where all 41 samples are homozygously inverted compared to the reference. In stark contrast, no misorientations are defined in the T2T-CHM13 genome, confirming its value as an improved reference (Additional file 1: Fig. S1B). Between the two references, inversion counts differ for most human chromosomes (*n* = 19) with the majority showing a net increase on T2T-CHM13 (*n* = 13) (Additional file 1: Fig. S2). Consistent with earlier analyses [7], Strand-seq detected the majority of large inversions (> 10 kbp) exclusively detecting 82 inversions with median size ~ 144 kbp and corresponding to ~ 62.7 Mbp of sequence (Additional file 1: Fig. S3A-D). Other technologies showed greater sensitivity for detecting smaller inversions (< 10 kbp), especially less than 1 kbp. While more genes are inverted by the larger inversions, smaller ones are particularly important for detecting rare gene-disruptive events (Additional file 1: Fig. S3E-F).

### Novel inversions and pericentromeric enrichment

We identify 63 sites of putative novel inversions with respect to T2T-CHM13, which improves overall sensitivity for inversion detection by ~ 21% (63/296) (Fig. 1A, Methods, Additional file 2: Table S2). Of these, 33 sites could be partially mapped to GRCh38 but share ≥ 90% overlap with nonsyntenic regions present in the T2T-CHM13 but not GRCh38 reference and, thus, potentially represent structural differences between the references. In addition, there are 12 sites that failed to map to GRCh38, the majority of which are small (< 1 kbp, *n* = 8). Nevertheless, two of these unmapped sites, one on chromosome 7 and one on chromosome 17, are ~ 206 kbp and ~ 1.38 Mbp in size and have 39% and 52% overlap with nonsyntenic regions, respectively. Lastly, we define 18 sites that both failed to map to GRCh38 and have ≥ 90% overlap with nonsyntenic regions and therefore are most likely novel (Additional file 1: Fig. S4). Almost all of the nonsyntenic regions where the new inversions map correspond to pericentromeric sequence in T2T-CHM13—defined here as sequence ± 1 Mbp adjacent to rDNA or satellite DNA. Indeed, we find that 20.6% (61/296) of inversion polymorphisms are pericentromeric (Fig. 1A, Methods). The effect is particularly pronounced on chromosomes 1, 2 and 7 where we observe a three to eightfold enrichment (*p* < 0.05, Permutation Test with Bonferroni multiple testing correction) (Fig. 1B, Methods, Additional file 1: Fig. S5, and Additional file 2: Table S3). Other chromosomes show more modest accumulation (i.e., chromosomes 9 and 16 with ~ 1.5-fold enrichments). We find 46% (28/61) of pericentromeric inversions associate with intrachromosomal SDs while another 26% (16/61) map to various classes of satellite DNA (Additional file 1: Fig. S6). As an example, we identify a ~ 1.3 Mbp inversion in the pericentromeric region of chromosome 1 that is completely absent from the GRCh38 reference (Fig. 1C). This large inversion represents the major allele in the human population (0.69 inverted allele frequency based on 82 analyzed haplotypes) (Fig. 1C). It is composed mostly of satellite repeats (human satellites [HSATs] and beta satellites) and we predict inversion breakpoints fall within or nearby HSAT repeats (Additional file 1: Fig. S7). We managed to confirm this inversion in four out of six Human Pangenome Reference Consortium (HPRC) assemblies of chromosome 1 centromere region (Additional file 1: Fig. S8) and found notable variability in inversion size and its distance to proximal alpha satellite repeat array (Additional file 1: Fig. S9). A second example includes a large 1 Mbp cluster of inversions mapping to the pericentromeric region of chromosome 7. In T2T-CHM13, this region is continuously assembled and contains six inverted loci that are either missing or likely misassembled in the GRCh38 reference [1]. The six satellite-associated inversions are polymorphic creating a diverse pattern of haplotypic structural diversity in the human population (Fig. 1D, Additional file 1: Fig. S10).

### Improved annotation of inversion polymorphisms

**Fig. 2:**
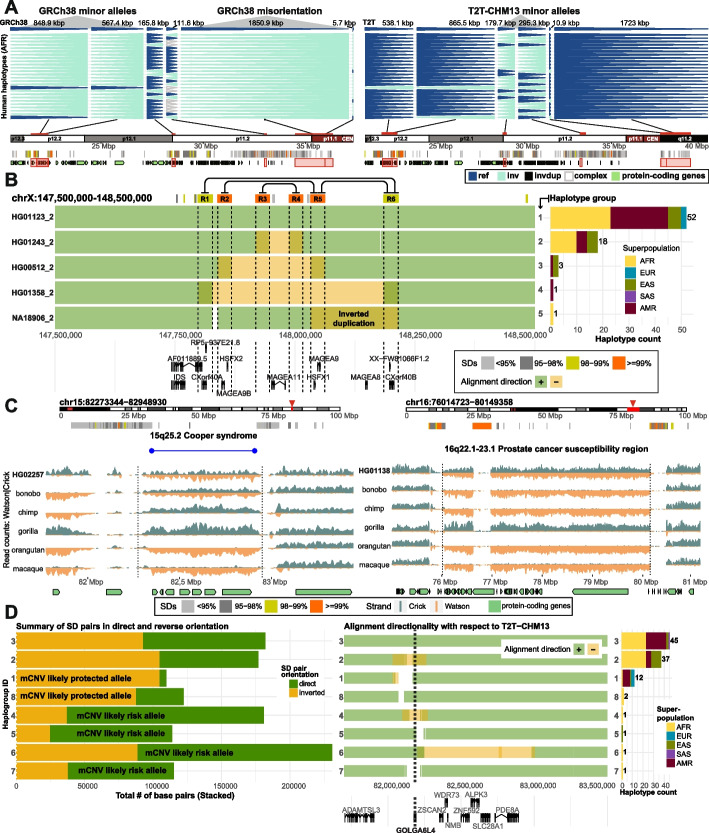
Improved representation of inversion polymorphisms in T2T-CHM13 and interpretation of TADs. **A** A “backgammon” plot for a 20 Mbp region at chromosome 16p depicting changes in the representation of major alleles as inverted (light blue) and direct (dark blue) orientation based on phased inversion genotypes reported with respect to GRCh38 and T2T-CHM13 reference genomes. Each horizontal set of arrowheads represents a single haplotype of African (AFR) ancestry. In most cases, GRCh38 was either erroneous or represented the minor allele. (See Additional file 1: Fig. S13 for all 82 haplotypes.) **B** Overlapping inversions on chromosome Xq28. Each row represents a unique human haplotype (haplotypes 1–5) of the Xq28 region visualized as a single human assembly aligned to T2T-CHM13 in forward ('+', green) or reverse ('-', orange) orientation. These aligned segments are displayed with respect to flanking segmental duplications (SDs) (R1-6) that likely mediate the inversions (connecting lines) and underlying protein-coding genes. We use transparency to convey positions of overlapping alignments, such as highlighted inverted duplication in haplotype 5. Barplot (right) shows the total counts of human haplotypes per haplotype group stratified by superpopulation. **C** Two disease-associated regions mapping to chromosomes 15q25.2 and 16q22.1–23.1 are depicted within chromosome-specific ideograms (red rectangle) with a zoom into the region flanked by SDs (colored horizontal bars) and pathogenic duplication and deletion breakpoints highlighted in blue and red horizontal lines, respectively. Strand-seq data highlight rare heterozygous inversions (see Fig. 1C for detailed description) discovered in a human sample with respect to the status in different nonhuman primate species. Homozygous inversions are orange while homozygous teal represents homozygous direct orientations. **D** Left plot summarizes the total number of base pairs for SD pairs in direct (dark green) and inverted (dark orange) orientatation for each haplogroup (in rows) marked as likely protected or at risk for morbid copy number variant (mCNV) formation. Middle plot shows unique human haplotypes (haplotypes 1–8) of the 15q25.2 region visualized as a single human assembly aligned to T2T-CHM13 in forward ('+', green) or reverse ('-', orange) orientation. Underlying protein-coding genes from this region are shown below. Barplot (right) shows the total counts of human haplotypes per haplotype group stratified by superpopulation

As mentioned above, we identified 26 regions where GRCh38 differed in orientation with respect to T2T-CHM13, but all 82 human haplotypes supported the T2T-CHM13 configuration (Additional file 1: Fig. S11 and Additional file 2: Table S4). While it is possible that these could be very low-frequency inversion polymorphisms, it is more likely that they simply represent misorientation errors. Many of these putative errors are large with median size of 16,306 bp (range: 488–2,346,462 bp). Excluding these likely misorientations, we find that T2T-CHM13 is much more likely to carry the major allele in the population. Specifically, we observe a threefold reduction of minor inversion alleles in T2T-CHM13 (*n* = 11) compared to GRCh38 (*n* = 33) (Additional file 1: Fig. S12). Because these regions contain or map near protein-coding genes (Additional file 1: Fig. S12 and Additional file 2: Table S4), these flips in orientation or changes in the major allele definition (Fig. 2A, Additional file 1: Fig. S13) can affect our interpretation of human genetic variation and functional annotation of the human genome. Such is the case for the inversion polymorphism affecting the melanoma antigen gene family cluster (*MAGE*) at the chromosome Xq28 region. In this region a minor (inverted) allele was originally reported in GRCh38 leading to the prediction of a series of nested inversions within a single haplotype [7]. However, with respect to T2T-CHM13, we can now report that the direct configuration represents the major allele (Additional file 1: Fig. S14). Rather than nested inversions, we observe four independent inversion events utilizing distinct SD pairs and affecting different *MAGE* genes at various frequencies in the human population. Analysis of HPRC phased genome assemblies (Fig. 2B, Methods) confirms five distinct human structural configurations that result in inversion polymorphisms of different sizes and frequencies among human populations with H5 predicted to be ancestral based on structural similarity to chimpanzee (Additional file 1: Fig. S15). Similarly, disease-associated regions, such as the 16p12.2 microdeletion region associated with neurodevelopmental disabilities [14, 15, 17], are now properly configured. This region was reported to be misoriented in the GRCh38 reference [18] and is now corrected within the T2T-CHM13 reference [1] (Additional file 1: Fig. S16) helping to better distinguish long and short inversions in this region. Finally, because proximity ligation experiments such as Hi-C depend on correct order and orientation of the assembled sequence, the correction of these errors can affect functional genome annotation. Such is the case when detecting topologically associated domains (TADs) at 16p12.2 that carry the long (GM20847) and short (HG02011) versions of an inversion at this locus. Here, the GRCh38 reference reports hard-to-interpret regional associations while T2T-CHM13 provides a much clearer picture of TADs that are in line with a presence of reported inversions (Methods, Additional file 1: Fig. S17).

### Discovery of novel rare inversion polymorphisms and disease-associated rearrangements

As part of a quality control assessment during the development of the first phase of the HPRC [8, 19], 10 additional Strand-seq datasets were generated from unrelated individuals for the HPRC. We applied these data to the T2T-CHM13 reference in an effort to discover additional rarer inversion polymorphisms. While the vast majority of inverted polymorphisms had been identified previously among the original 41 samples, we did identify five additional inversions (Additional file 2: Table S5), including a novel structural configuration for the Xq28 *MAGE* gene cluster described above. The other novel inversions include large > 1 Mbp events corresponding to chromosomes 15q25.2, 16p11.2, 16q22.1–23.1 and 22q11.21 (Fig. 2C). Notably, all but one of these rare inversion polymorphisms overlap a pathogenic CNV in the human population, strengthening our recent observation of disease association [7]. This includes a large inversion polymorphism encompassing one of the most common rearrangements associated with autism at chromosome 16p11.2 and one of the most frequent deletions in the human population at the DiGeorge/VCF syndrome critical region (Additional file 1: Fig. S18) [20, 21]. We further investigate the Cooper syndrome region on chromosome 15q25.2 using available HPRC assemblies and define eight structurally diverse haplotypes with various frequencies in human populations. We predict haplogroups 1 and 8 are likely to be protected while haplogroups 4–7 are likely at increased risk of microdeletion/duplication of the 15q25.2 region due to higher number of SD bases in direct orientation (Fig. 2D, Additional file 1: Fig. S19). We successfully characterized the SD-associated inversion breakpoints of the inverted haplotype corresponding to haplogroup 6 with an inversion breakpoint falling within the ~ 5 kbp region of nearly perfect homology (99.8% identical) (Methods, Additional file 1: Fig. S20). Lastly, we report a massive ~ 4.13 Mbp inversion located at chromosome 16q22.1–23.1, a region previously linked to prostate cancer [22] where, to our knowledge, no inversion has yet been identified. Comparison to nonhuman primate data [6] and previous studies [23] suggests that for three out of five events the rare, inverted configuration in the human population represents the ancestral orientation (Fig. 2C).

Previous studies have highlighted an increase in both specificity and sensitivity for single-nucleotide variant and CNV detection when using the T2T-CHM13 reference in lieu of GRCh38 [2, 3]. Our results suggest the effect is the most pronounced for the discovery and characterization of inversion polymorphisms. The 21% improvement in discovery stems in large part from the fact that inversions most strongly associate with repetitive DNA [24–26] and the addition of these previously inaccessible regions allows for their discovery by the mapping of Strand-seq data to these regions for the first time. In addition to these new discoveries, we provide further evidence that the T2T-CHM13 reference better represents the orientation of the major allele and identify 26 relatively large misorientations (total of 6.4 Mbp of sequence) in the original GRCh38 reference genome that have persisted for many earlier iterations of the human reference genome (Additional file 1: Fig. S21).

Our analysis also revealed a greater propensity of polymorphic inversions to cluster within pericentromeric regions. However, this may not be surprising given that pericentromeric regions have been known for more than two decades to be hotspots for the accumulation of high-identity SDs [27–30]. Intrachromosomal SDs, in particular, drive the formation of many of the largest inversions via non-allelic homologous recombination and, indeed, nearly 50% of the pericentromeric inversions in this study have intrachromosomal SD pairs delineating their breakpoints (Additional file 1: Fig. S6). Of note, we also observe a relatively high proportion (16%) of satellite-associated inversion polymorphisms, especially within selected pericentromeric regions where they appear to cluster creating considerable haplotypic diversity (Fig. 1C,D). Polymorphic inversions have the capacity to reduce recombination [31] and one possibility for reduced recombination across centromeres may be that the enrichment of such pericentromeric inversions at their flanks interferes with synapsis during meiosis. Alternatively, the reduced recombination may predate these structural features and instead promote the accumulation of large repeats promoting unequal crossover and inversion formation.

Finally, our investigation of 10 additional human genomes from the HPRC [8] highlights the value of continued inversion polymorphism discovery. As previously reported [7], the number of new inversions discovered is predictably low when compared to other forms of human variation due to an excess of common variation for this class of variant in the human population. Nevertheless, the additional rare polymorphisms we identified are > 1 Mbp in length, spanning large swaths of genes and overlapping regions of genomic instability related to human disease. In particular, we recently demonstrated a fivefold association of recurrent inversion polymorphisms with recurrent genomic disorders among children with neurodevelopmental disorders [7]. One hypothesis is that the recurrent inversions are reshaping the architecture of the flanking SDs creating both protective and predisposed haplotypes to rearrangement as has been shown for a few loci [13, 32, 33]. To address this, it will be critical to survey many more human genomes and to sequence resolve the large complex SDs flanking the inversion polymorphisms. Currently, methods such as trio-hifiasm fail to fully sequence resolve the many complex flanking SD regions or, in some cases, do not even identify the large inversion polymorphisms based on sequence assembly only. New methods, however, such as Verkko [34], that couple both HiFi (high-fidelity PacBio) and ultra-long ONT (Oxford Nanopore Technologies) data show considerable promise in resolving a greater fraction of these regions.

## Conclusions

We describe the most comprehensive evaluation of inversion polymorphisms with respect to the recently released complete sequence of the human genome (T2T-CHM13). We report an improved inversion discovery in previously inaccessible regions such as a large ~ 1.3 Mbp pericentromeric inversion on chromosome 1 and a peculiar cluster of pericentromeric inversions on human chromosome 7. T2T-CHM13 corrects a number of longstanding inversion polymorphisms that have persisted in the previous human reference assemblies. We find that the T2T reference is three times more likely to represent the correct orientation of the major human allele. Lastly, we demonstrate the value of continuous inversion discovery among diverse human populations by reporting four rare and large inversions at chromosomes 15q25.2 (~ 675 kbp), 16p11.2 (~ 1.38 Mbp), 16q22.1–23.1 (~ 4.13 Mbp) and 22q11.21 (~ 2.25 Mbp)—all genome instability regions associated with disease. Mapping to the T2T reference improves our understanding of inversion polymorphism more than any other class of genetic variants making T2T-CHM13 the preferential reference for future investigations into such human genetic variation in diverse human populations—one of the goals of the HPRC [35].

## Methods

### Strand-seq data generation and data processing

Strand-seq data were generated as follows. EBV-transformed lymphoblastoid cell lines HG01138 and HG01888 (from the 1000 Genomes Project) were obtained from Coriell Institute and cultured with BrdU (100 uM final concentration; Sigma, B9285) for 18 or 24 h, and single isolated nuclei (0.1% NP-40 lysis buffer [36]) were sorted into 96-well plates using the BD FACSMelody cell sorter. In each sorted plate, 94 single cells plus one 100-cell positive control and one 0-cell negative control were deposited. Strand-specific single-cell DNA sequencing libraries were generated using the previously described Strand-seq protocol [36, 37] and automated on the Beckman Coulter Biomek FXp liquid handling robotic system [38]. Following 15 rounds of PCR amplification, 288 individually barcoded libraries (amounting to three 96-well plates) were pooled for sequencing on the Illumina NextSeq 500 platform (MID-mode, 75 bp paired-end protocol). The demultiplexed FASTQ files were aligned to the T2T-CHM13 reference assembly (v1.0 and v1.1) using BWA (version 0.7.15–0.7.17) for standard library selection. Aligned reads were sorted by genomic position using SAMtools (version 1.10) and duplicate reads were marked using sambamba (version 1.0). Low-quality libraries were excluded from future analyses if they showed low read counts (< 50 reads per Mbp), uneven coverage, or an excess of ‘background reads’ (reads mapped in opposing orientation for chromosomes expected to inherit only Crick or Watson strands) yielding noisy single-cell data, as previously described [36]. All cell lines used in this study were from the 1000 Genomes Project and were authenticated by mapping Strand-seq data to genome assemblies.

### Generation of inversion callset with respect to the T2T-CHM13 reference (v1.0)

In this study we applied the same multi-platform-based inversion discovery procedure as reported recently [7]. This procedure involves independent inversion discovery using PAV (long-read-based phased assemblies), Strand-seq (strand-specific short-read sequencing), and Bionano Genomics (optical mapping). We note that the inversion callset based on Strand-seq and Bionano Genomics underwent extensive manual curation in order to ensure high accuracy of a final inversion callset. Subsequently, independent inversion callsets were merged into a nonredundant inversion callset using SV-pop [39, 40].

### Lifting inversion callset to the latest version of T2T-CHM13 reference (v1.1)

Since the original inversion callset was done with respect to T2T-CHM13 v1.0, we decided to lift coordinates to v1.1 using liftOver. The only differences between v1.0 and v1.1 were the addition of missing rDNA and improved polishing within telomeres. To report inversion coordinates with respect to the latest version of T2T-CHM13 (v1.1, only difference with v2.0 is an addition of chromosome Y) reference, we used a command line version of UCSC liftOver tool (liftOver {input.bed} {input.chain} {output.bed} {output.unmapped}). We used publicly available liftOver chains ‘v1.0_to_v1.1.chain’ at https://s3-us-west-2.amazonaws.com/human-pangenomics/index.html?prefix=T2T/CHM13/assemblies/changes/v1.0_to_v1.1/. We attempted to lift all 374 detected sites. Of those, only one site genotyped as an inverted duplication (‘chr14-2,842,055-INV-181339’) positioned in chr14 rDNA failed to lift. Importantly, all sites (*n* = 296) genotyped as balanced inversions were successfully lifted to T2T-CHM13 (v1.1/v2.0) coordinates. These coordinates will be used for all analyses reported in this paper (Additional file 2: Table S1). Similarly, we used liftOver chains to translate coordinates from T2T-CHM13 to GRCh38, as was done for a complex region on chromosome 7 reported in Fig. 1D; liftOver chain from T2T-CHM13 v2.0 to GRCh38 was downloaded from https://s3-us-west-2.amazonaws.com/human-pangenomics/index.html?prefix=T2T/CHM13/assemblies/chain/v1_nflo/chm13v2-grch38.chain.

### Mapping inversion coordinates to GRCh38

To translate coordinates of GRCh38 inversions into the T2T-CHM13 coordinate space, we decided to extract FASTA sequence from each inverted region and try to map such FASTA sequence onto the T2T-CHM13 reference using minimap2 [41]. This is because breakpoints of many inversions lie within SDs, which makes simple lifting of coordinates using liftOver difficult and results in many inverted regions to fail to lift. We mapped FASTA sequence extracted from inverted regions in GRCh38 to the T2T-CHM13 reference using minimap2 (version 2.24) with following parameters: -secondary = no –eqx -ax asm20. We filtered out alignments with mapping quality zero and alignments mapped to a different chromosome than the one the FASTA sequence was extracted from. Inverted regions divided in multiple mappings were collapsed together, such that distance between subsequent mappings were no longer than 100 kbp. Lastly, we excluded mapped and collapsed ranges where the size was more than 50% larger or smaller than the original inversion range. Using this procedure, we were able to map 266 of all 296 balanced inversions in the T2T-CHM13 callset. The same procedure was used when mapping inversion coordinates from GRCh38 to T2T-CHM13.

### Definition of likely novel inversions in T2T-CHM13

To define likely novel inversions detected with respect to T2T-CHM13, we set to investigate mappings of T2T-CHM13 inverted regions onto the GRCh38 reference (see section above) as well as previously defined nonsyntenic regions between T2T-CHM13 and GRCh38. The annotation of nonsyntenic regions in T2T-CHM13 with respect to GRCh38 was taken from the previous study [3]. We calculated the percent overlap between T2T-CHM13 inversions (*n* = 296) and the list of nonsyntenic regions. Inversions with ≥ 90% with the nonsyntenic regions were deemed as ‘nonsyntenic’ because their structure and relative orientation between T2T-CHM13 and GRCh38 might differ (Additional file 1: Fig. S4). We marked inverted sites reported as nonsyntenic that also failed to map onto the GRCh38 reference as likely novel inversions in T2T-CHM13.

### Analysis of pericentromeric inversions

To define if an inversion lies within a pericentromeric regions of the T2T-CHM13 assembly, we took a recently released annotation of centromeric repeats from https://s3-us-west-2.amazonaws.com/human-pangenomics/index.html?prefix=T2T/CHM13/assemblies/annotation/chm13.draft_v1.1.cenAnnotation.bed. Pericentromeric regions were defined as continuous regions that include HSATs, alpha satellites, and rDNA with 1 Mbp of extra sequence at its flanks. Inversions that overlap (at least one base pair) with defined pericentromeric regions are considered as ‘pericentromeric’. Next, we used the R package regioneR [42] with its function ‘permTEST’ to perform permutation testing (*n* = 1,000 permutations) of pericentromeric inversions per chromosome. At each permutation, we randomized the position of each inversion per chromosome using regioneR’s function ‘randomizeRegions’ such that each inversion is assigned a random position along a given chromosome at each permutation. At each permutation, we counted the number of inversions overlapping with the pericentromeric region of any given chromosome. Due to multiple testing, we adjusted resulting p-values using Bonferroni correction. Subsequently, we evaluated the sequence composition of each inversion from pericentromeric regions (*n* = 61). To do this, we calculated overlap of inverted bases with a set of genomic features, such as HSATs, beta satellites, alpha satellites, monomeric regions, rDNA, and SD pairs. SD pairs were defined as intrachromosomal SDs that are no further apart than 5 Mbp. Inverted bases that do not overlap any of the above listed features were marked as ‘other’.

### Extraction of FASTA sequence from a region of interest (ROI)

To extract FASTA sequence from an ROI in T2T-CHM13 coordinates, we aligned available human assemblies from HPRC and Human Genome Structural Variation Consortium (HGSVC) datasets to the T2T-CHM13 (v1.1) reference using minimap2 (version 2.24) with the following parameters: ‘-x asm20 –secondary = no -s 25000’. Note, an increased value was used for parameter -s in order to filter out less contiguous alignments. Next, we used rustybam (version 0.1.27, 10.5281/zenodo.5875012) and its functionality called ‘liftover’ in order to subset alignments in PAF format to a desired ROI. Then we used such subsetted PAF file(s) in order to extract the query FASTA sequence using R package SaaRclust [43] and its function ‘regions2FASTA’.

### Minor allele detection and misorientation validation

First, we mapped balanced inversions and putative misorientations in GRCh38 coordinates (*n* = 330) to T2T-CHM13 using the procedure described above. Of the total 330 regions, 311 (281 balanced inversions and 30 misorientations) were successfully mapped to T2T-CHM13 coordinates. Next, we calculated the fraction of Watson (minus, negative strand) and Crick (plus, positive strand) reads mapped to each region in GRCh38 and T2T-CHM13 coordinates across all unrelated samples (*n* = 41) used in this study. We required that each evaluated site include at least 20 mapped Strand-seq reads in both reference coordinates. Subsequently, a minor allele was defined as a region where there is at least 25% difference between a fraction of Watson and Crick reads mapped to GRCh38 and T2T-CHM13 for any given region. Also, we required that the ratio of Watson and Crick reads with respect to both references is no more than 25% different. The minor allele in GRCh38 is reported if the fraction of Crick reads (plus reads) is smaller than the fraction of Crick reads in T2T-CHM13 over the same region. This means that the majority of reads map in minus orientation across all unrelated samples while the majority of reads with respect to T2T-CHM13 map in direct (plus) orientation. Minor alleles in T2T-CHM13 were defined in an opposite manner as sites with the majority of reads mapped in minus orientation, while for the same region, GRCh38 counts mostly plus reads.

### Hi-C data analysis and visualization

To visualize Hi-C data, we first aligned short paired-end reads to the reference genome of interest (T2T-CHM13, v1.1). For this we used BWA (version 0.7.17) [44] as follows: `bwa mem -5SP {input.ref} {input.pair1} {input.pair2}`. Note that set parameters -5SP are recommended to use only for the alignment of Hi-C data in order to obtain better alignments that are usually further apart than in standard mate-pair libraries. After the alignment we mark duplicate reads using `sambamba markdup` [45] and sorted by query name as is standard for Hi-C analysis pipelines. Such aligned BAM files were processed using R package diffHic [46]. First, we used the diffHic function ‘preparePairs’ in order to read in Hi-C alignments. At this step we filtered out reads with mapping quality less than 10 as well as any duplicate reads. Next, we used the diffHic function called ‘squareCounts’ in order to count the number of Hi-C interactions between two genomic bins of user-defined size. Lastly, the level of genomic interactions was visualized as diagonal squares colored by continuous heatmap colors on log scale.

### Detecting novel inversions using Strand-seq

In this study we added 10 additional samples where we called inversions using Strand-seq only [25, 47]. A novel inversion was defined as an inverted site not detected among the 41 samples used to generate the main inversion callset with respect to T2T-CHM13. Each newly detected inversion was checked for support using Strand-seq data from nonhuman primates to evaluate the ancestral state of a given locus. Each newly detected inversion shows change in orientation in at least one nonhuman primate.

### Genome structural diversity of Xq28 and 15q25.2 regions

We start by extracting FASTA sequence from a desired ROI as described in section ‘Extraction of FASTA sequence from a region of interest’. We select only assembled contigs with a complete span of the ROI such that contig boundaries are no further than 100 kbp from left and right ROI coordinates. We reverse complement assembly FASTA sequence in case the first and last contig alignments of at least 50 kbp are in minus orientation with respect to the reference. This is done to synchronize orientation among all FASTA files. We align such FASTA files from the ROI with respect to the reference (T2T-CHM13, v1.1) using minimap2 (version 2.24, parameters: -x asm20 –secondary = no -c –eqx -r 500,10 k) in order to evaluate alignment directionality. In the case of the 15q25.2 region, we also align each FASTA file to itself using minimap2 (version 2.24, parameters: ‘-x asm10 -c –eqx -D -P –dual = yes -r 10,50’) to record a relative orientation (reverse or direct) of self-alignments within each FASTA file. Lastly, we select self-alignments that are at least 500 kbp apart and calculate the fraction and the total length of these self-alignments stratified by their relative orientation. We then use this information as a proxy to predict if an intervening region (between flanking self-alignments) is likely predisposed to an inversion or a CNV. Note that we have adjusted minimap2 parameter -r in order to decrease self-alignment redundancy as well as to obtain continuous alignments over short inverted regions (see minimap2 manual page for more details).

### Inversion breakpoint mapping

To map inversion breakpoints of a defined inversion at the 15q25.2 region, we selected the FASTA sequence of an inverted haplotype (HG02257_1) and direct haplotype represented by T2T-CHM13 reference corresponding to region chr15:81700000–83500000. Next, we aligned both the inverted and direct haplotypes to themselves using minimap2 (version 2.24) in order to define pairs of identical sequences (SDs) within each haplotype. We selected only those pairs that were at least 500 kbp distance in order to obtain only those pairs that flank the inverted region (~ 675 kbp in size). We further selected those pairs that are in an inverted orientation with respect to each other. Lastly, we extracted FASTA sequence from such SD pairs for both inverted and direct haplotypes and continued with inversion breakpoint mapping as described in Porubsky et al. (2022) [8].

## Supplementary Information

**Additional file 1: Figure S1.** T2T-CHM13 inversion callset summary and comparison to GRCh38 (*n* = 373). **Figure S2.** Differences between GRCh38 and T2T-CHM13 callsets. **Figure S3.** Inversion callset summary with respect to T2T-CHM13 reference. **Figure S4.** Nonsyntenic and likely novel sites in T2T-CHM13 inversion calls. **Figure S5.** Enrichment of inversions in pericentromeric regions. **Figure S6.** Sequence composition of inversions from pericentromeric regions. **Figure S7.** Novel pericentromeric inversion on chromosome 1. **Figure S8.** Complete assemblies of chromosome 1 centromeric region. **Figure S9.** Relative position of alpha satellite array and novel pericentromeric inversion on chromosome 1. **Figure S10.** Inversion phasing at pericentromeric region of chromosome 7. **Figure S11.** Evaluation of putative misorients in GRCh38 with respect to T2T-CHM13. **Figure S12.** Evaluation of inversion differences between GRCh38 and T2T-CHM13 references. **Figure S13.** Examples of minor and misoriented alleles at chromosome 16. **Figure S14.** Structural differences at Xq28 between GRCh38 and T2T-CHM13. **Figure S15.** Diverse structural haplotypes at the Xq28 region. **Figure S16.** Structural differences at 16p12.2 between GRCh38 and T2T-CHM13. **Figure S17.** Topological differences at 16p12.2 between GRCh38 and T2T-CHM13. **Figure S18.** Rare inversions at disease relevant loci. **Figure S19.** Diverse structural haplotypes at 15q25.2 region. **Figure S20.** Assembled inversion breakpoints at 15q25.2 and inversion breakpoint mapping. **Figure S21.** Example of long-lasting misorientation errors in previous human genome references. **Supplementary Notes. **Consortia.

**Additional file 2: Table S1.** Nonredundant inversion callset reported in this study. **Table S2.** Putative novel inversions with respect to T2T-CHM13 reference. **Table S3.** Enrichment of inversions in pericentromeric regions. **Table S4.** List of minor alleles and resolved orientation errors in GRCh38. **Table S5.** Novel inversions in HPRC Strand-seq dataset.

**Additional file 3.** Review history.

## Data Availability

HPRC assemblies used in this study have been reported by Liao et al. (2022) [19]. HGSVC assemblies used in this study have been reported by Ebler et al. (2022) [48]. Strand-seq data for eight samples (HG01123, HG01258, HG01358, HG01361, HG01891, HG02257, HG02486, and HG02559) reported by Porubsky et al. (2022) [8] as well as additional samples, HG01138 and HG01888, used in this study can be obtained via ENA accession number: PRJEB54100 [49]. Hi-C data for two samples (HG02011 and NA20847) presented in this study can be obtained at accession number: ERP123231 [50]. Additional data, including custom R functions and scripts used in this study, can be obtained at Zenodo (10.5281/zenodo.7742689) [51] and on GitHub (https://github.com/daewoooo/T2T-CHM13_inversion_polymorphism_paper) [52] under MIT license.
