# Supplementary material for: Inversion polymorphism in a complete human genome assembly
Source: Genome Biol. 2023 Apr 30;24:100. doi: 10.1186/s13059-023-02919-8 (PMC10150506; doi:10.1186/s13059-023-02919-8)
Supplement: Supplementary file 1 — Additional file 1: Figure S1. T2T-CHM13 inversion callset summary and comparison to GRCh38 (n = 373). Figure S2. Differences between GRCh38 and T2T-CHM13 callsets. Figure S3. Inversion callset summary with respect to T2T-CHM13 reference. Figure S4. Nonsyntenic and likely novel sites in T2T-CHM13 inversion calls. Figure S5. Enrichment of inversions in pericentromeric regions. Figure S6. Sequence composition of inversions from pericentromeric regions. Figure S7. Novel pericentromeric inversion on chromosome 1. Figure S8. Complete assemblies of chromosome 1 centromeric region. Figure S9. Relative position of alpha satellite array and novel pericentromeric inversion on chromosome 1. Figure S10. Inversion phasing at pericentromeric region of chromosome 7. Figure S11. Evaluation of putative misorients in GRCh38 with respect to T2T-CHM13. Figure S12. Evaluation of inversion differences between GRCh38 and T2T-CHM13 references. Figure S13. Examples of minor and misoriented alleles at chromosome 16. Figure S14. Structural differences at Xq28 between GRCh38 and T2T-CHM13. Figure S15. Diverse structural haplotypes at the Xq28 region. Figure S16. Structural differences at 16p12.2 between GRCh38 and T2T-CHM13. Figure S17. Topological differences at 16p12.2 between GRCh38 and T2T-CHM13. Figure S18. Rare inversions at disease relevant loci. Figure S19. Diverse structural haplotypes at 15q25.2 region. Figure S20. Assembled inversion breakpoints at 15q25.2 and inversion breakpoint mapping. Figure S21. Example of long-lasting misorientation errors in previous human genome references. Supplementary Notes. Consortia. [file 13059_2023_2919_MOESM1_ESM.pdf]

Inversion polymorphism in a complete human genome assembly

David Porubsky[1], William T. Harvey[1], Allison N. Rozanski[1], Jana Ebler[2], Wolfram Höps[3], Hufsah Ashraf[2], Patrick Hasenfeld[3], Human Pangenome Reference Consortium (HPRC)*, Human Genome Structural Variation Consortium (HGSVC)*, Benedict Paten[4], Ashley D. Sanders[5], Tobias Marschall[2], Jan O. Korbel[3,6], Evan E. Eichler[1,7]

1. Department of Genome Sciences, University of Washington School of Medicine, Seattle, WA, USA.
2. Heinrich Heine University, Medical Faculty, Institute for Medical Biometry and Bioinformatics, Moorenstraße 5, 40225 Düsseldorf, Germany.
3. European Molecular Biology Laboratory (EMBL), Genome Biology Unit, Meyerhofstr. 1, 69117 Heidelberg, Germany.
4. UC Santa Cruz Genomics Institute, University of California Santa Cruz, Santa Cruz, CA, USA.
5. Berlin Institute for Medical Systems Biology, Max Delbrück Center for Molecular Medicine in the Helmholtz Association, Berlin, Germany.
6. European Molecular Biology Laboratory, European Bioinformatics Institute, Wellcome Genome Campus, Hinxton, Cambridge, CB10 1SD, United Kingdom.
7. Howard Hughes Medical Institute, University of Washington, Seattle, WA, USA.

Corresponding author: eee@gs.washington.edu

SUPPLEMENTARY FIGURES (S1-S21):

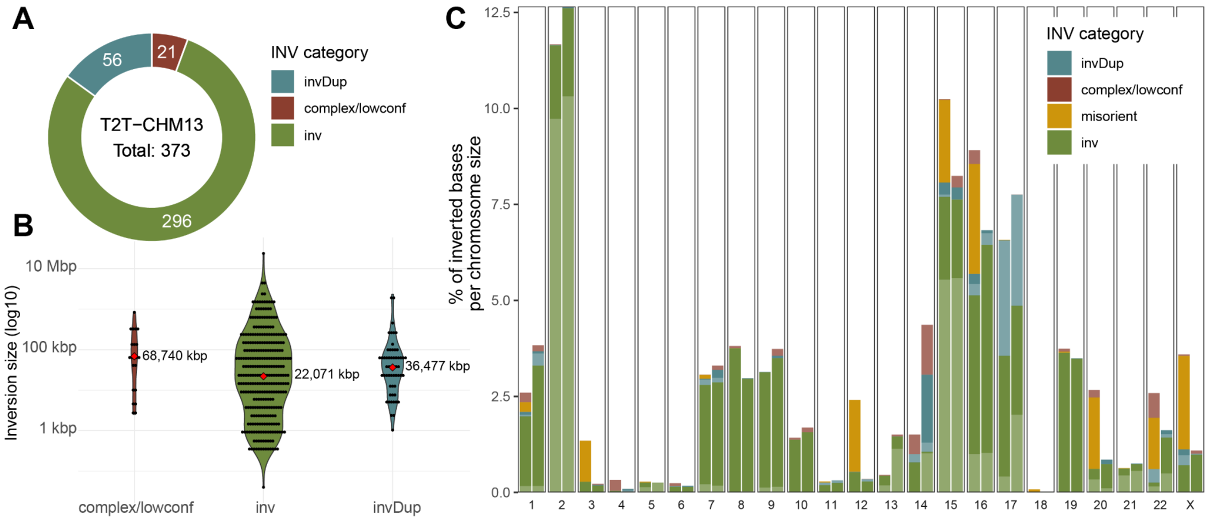

Figure S1: T2T-CHM13 inversion callset summary and comparison to GRCh38 (n=373).

A) A donut plot showing the total counts of inversion classes ('complex/lowconf' - complex and low-confidence calls, 'inv' - balanced inversions, and 'invDup' - inverted duplications) defined based on Arbigent and PAV genotypes [7]. **B**) A size distribution of the whole callset stratified per inversion class. **C**) A barplot showing the percentage of inverted bases per inversion category (misorient - misorientation, inv - balanced inversion, invDup - inverted duplication, and complex/lowconf - structurally complex region or low-confidence call) given the chromosome size. For each chromosome, the left- and right-side bars represent the fraction of inverted bases in GRCh38 and T2T-CHM13, respectively. Lighter color highlights bases being inverted only in a single sample (see light green color for chromosome 2 contributed by a single pericentromeric inversion ~23 Mbp in size in sample NA19650).

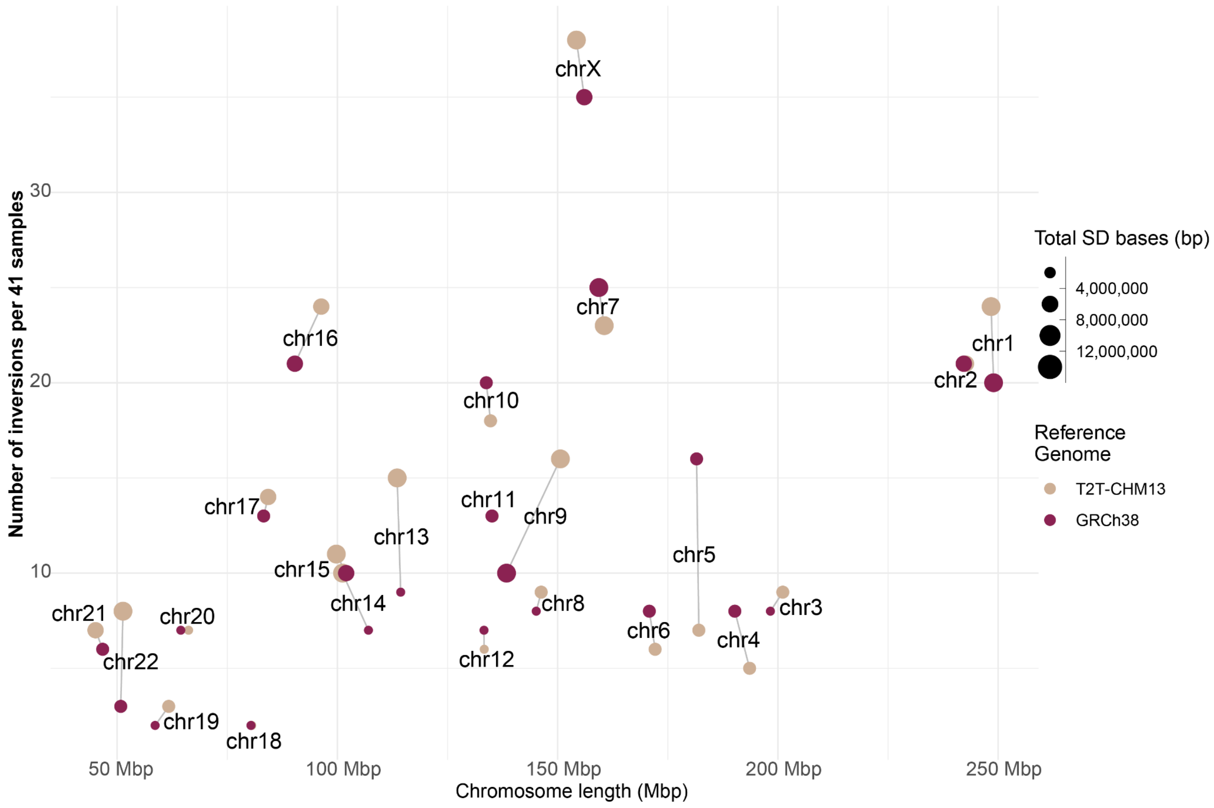

Figure S2: Differences between GRCh38 and T2T-CHM13 callsets.
A scatterplot shows the total number of balanced inversions detected per chromosome (y-axis) given the chromosome length (x-axis) separately per GRCh38 (beige) and T2T-CHM13 (purple) inversion callset. Size of each dot represents the total number of SD bases reported for a given chromosome and given reference.

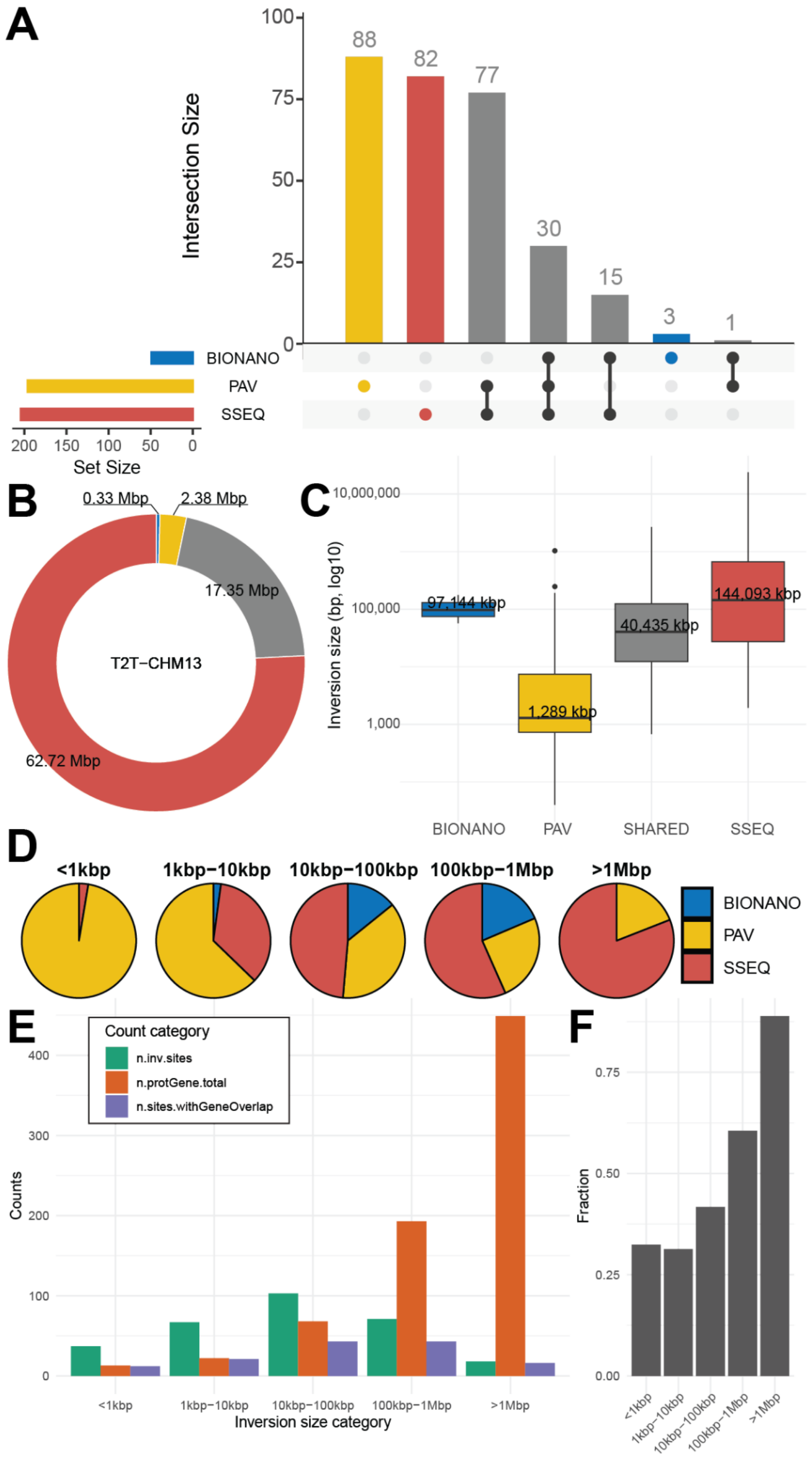

Figure S3: Inversion callset summary with respect to T2T-CHM13 reference.
A) An upset plot showing the total number of inversions uniquely detected by each technology ('PAV' - phased-assembly; 'BIONANO' - Bionano Genomics, and 'SSEQ' - Strand-seq) and those detected by two and more (gray). **B**) A donut plot showing the number of megabases contributed separately by a single technology and those supported by more than one (gray). **C**) A size distribution of balanced inversions stratified by technology supporting each inverted site and those supported by more than one technology (gray - SHARED). **D**) Pie charts show proportions of balanced inversions (n=296), supported by each evaluated technology (see above) stratified per size. **E**) A barplot stratifying balanced inversions by size (x-axis) and for each size category showing the number of inversions (green), total number of overlapping protein-coding genes (orange), and number of inversion overlapping at least one protein-coding gene (purple). **F**) Fraction of inverted sites in each size category that overlap with at least one protein-coding gene.

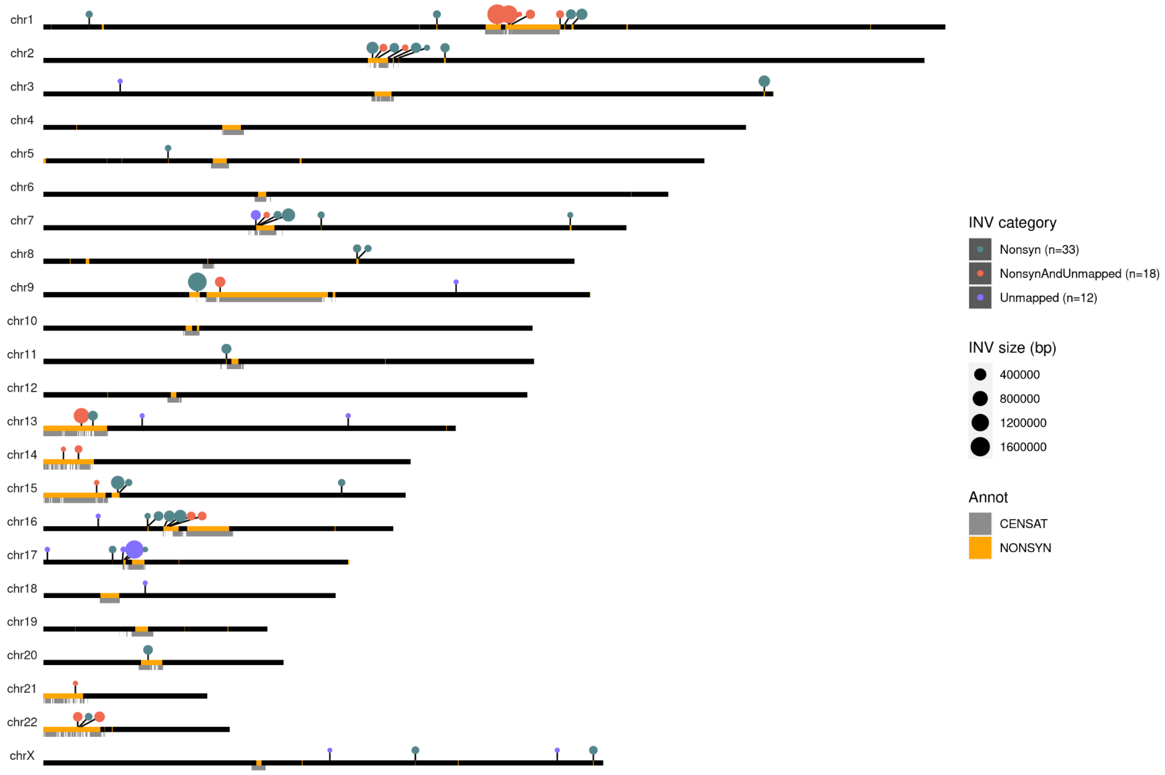

Figure S4: Nonsyntenic and likely novel sites in T2T-CHM13 inversion calls.
The ideogram shows the position and size (dot size) of all balanced inversions (n=63) that either fall within (≥90% reciprocal overlap) nonsyntenic regions between GRCh38 and T2T-CHM13 ('Nonsyn') or failed to map to GRCh38 reference ('Unmapped') (**Methods, Additional file 2: Table S2**). Red dots point to regions whose sequence failed both to map to GRCh38 reference and fall within nonsyntenic regions (n=18).

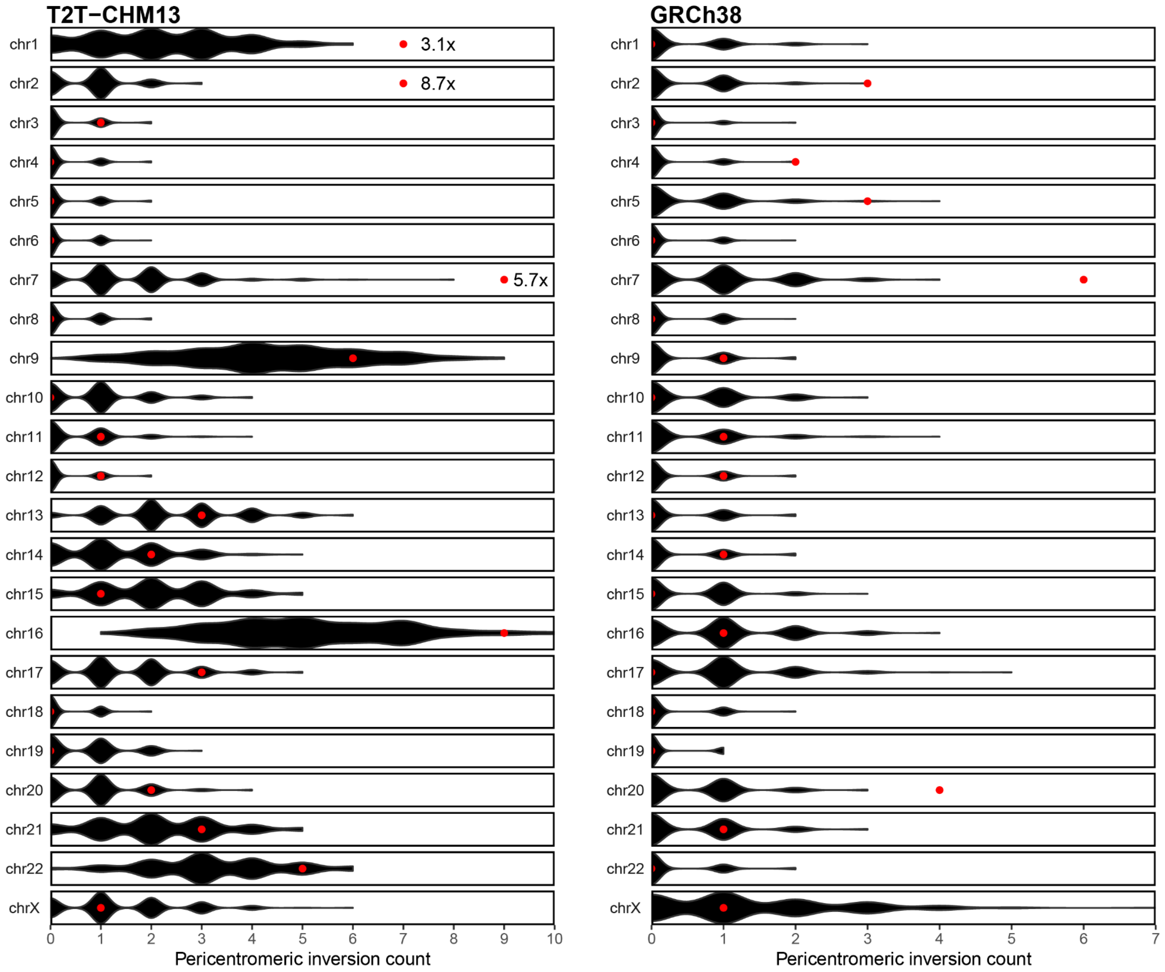

Figure S5: Enrichment of inversions in pericentromeric regions.

Enrichment analysis was done using a permutation test by counting the number of inversions overlapping with pericentromeric regions separately per each chromosome. Permuted counts of pericentromeric inversions are shown as black violin plots. Observed values are shown as red dots. Enrichment analysis is reported separately with respect to T2T-CHM13 (left) and GRCh38 (right) (**Methods**). For the T2T-CHM13 reference, we highlight fold-enrichment values for chromosomes that reached significance after Bonferroni correction (**Additional file 2: Table S3**).

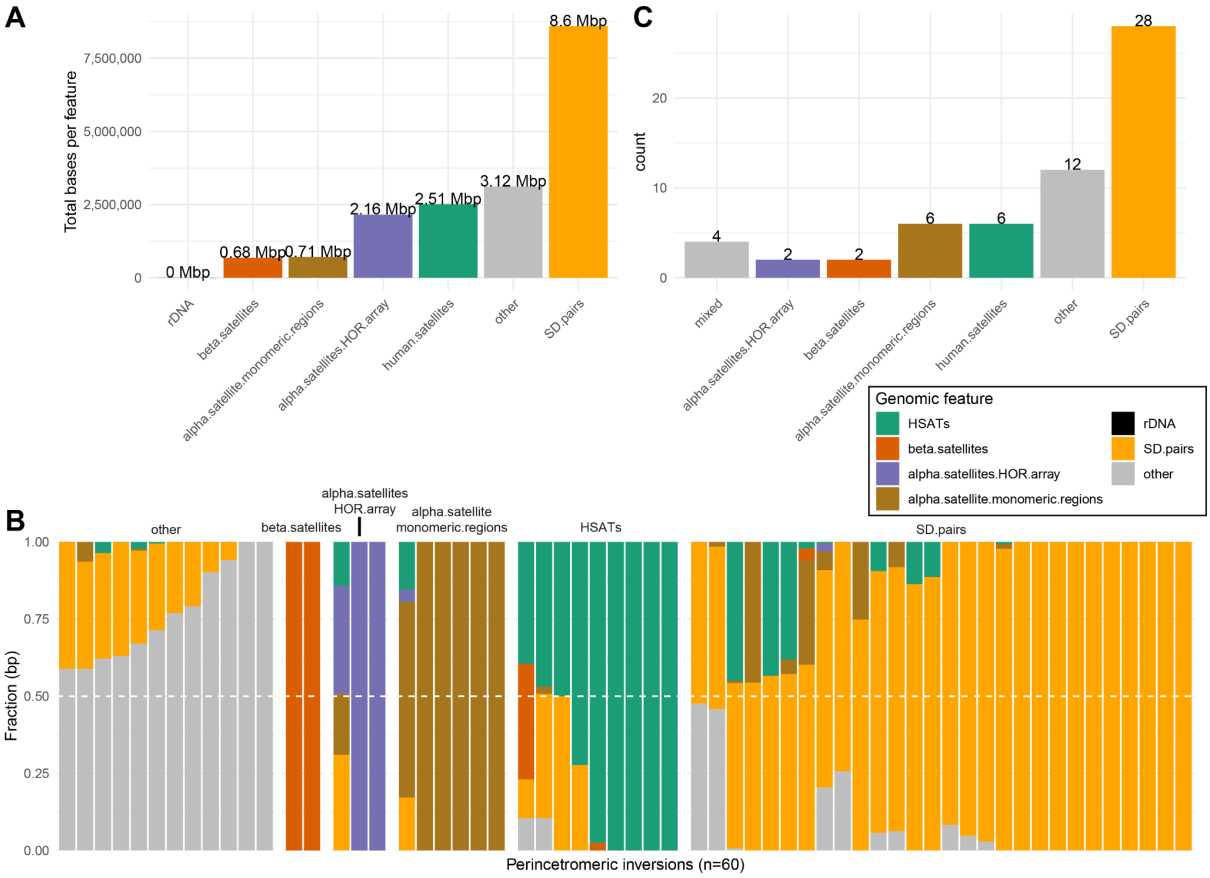

Figure S6: Sequence composition of inversions from pericentromeric regions.
A) The total number of base pairs of various genomic features (such as various classes of human satellites, 'SD.pairs' - intrachromosomal pairs of SDs no further than 5 Mbp apart and ‘other’ - none of these features) overlapping pericentromeric inversions (n=60). **B**) Proportion of genomic features assigned to each brnn region based on the number of ‘burned’ haplotypes within each brnn region. **C**) An assignment of each pericentromeric inversion to a single feature based on the majority overlap (proportion of the given feature >0.5) or are labeled as ‘mixed’ if no feature is >0.5.
NOTE: In this analysis we excluded the large pericentromeric inversion on chromosome 2 that is ~23 Mbp in size to prevent our results being skewed by including such a large genomic region.

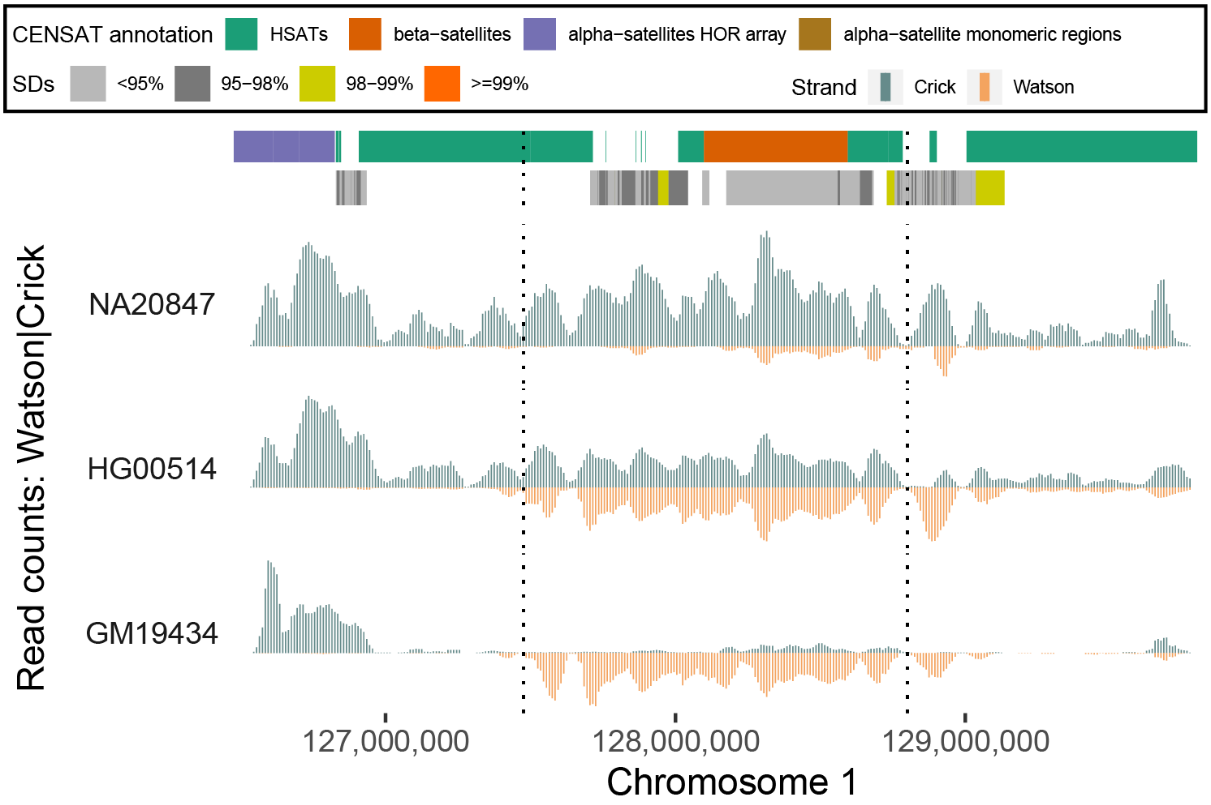

Figure S7: Novel pericentromeric inversion on chromosome 1.
A zoomed-in plot shows the novel pericentromeric inversion on chromosome 1 (highlighted by dotted lines) presented in Fig. 1C. The read-coverage profiles of Strand-seq data over a chromosome 1 centromeric region are summarized as binned (bin size: 50 kbp step size: 10 kbp) read counts represented as bars above (teal; Crick read counts) and below (orange; Watson read counts) the midline. Dotted lines highlight the novel centromeric inversion detected on chromosome 1 only with respect to T2T-CHM13. In this region equal coverage of Watson and Crick counts represents a heterozygous inversion as only one homologue is inverted with respect to the reference while reads aligned only in Watson orientation represents a homozygous inversion. Above is a centromere and SD annotation.

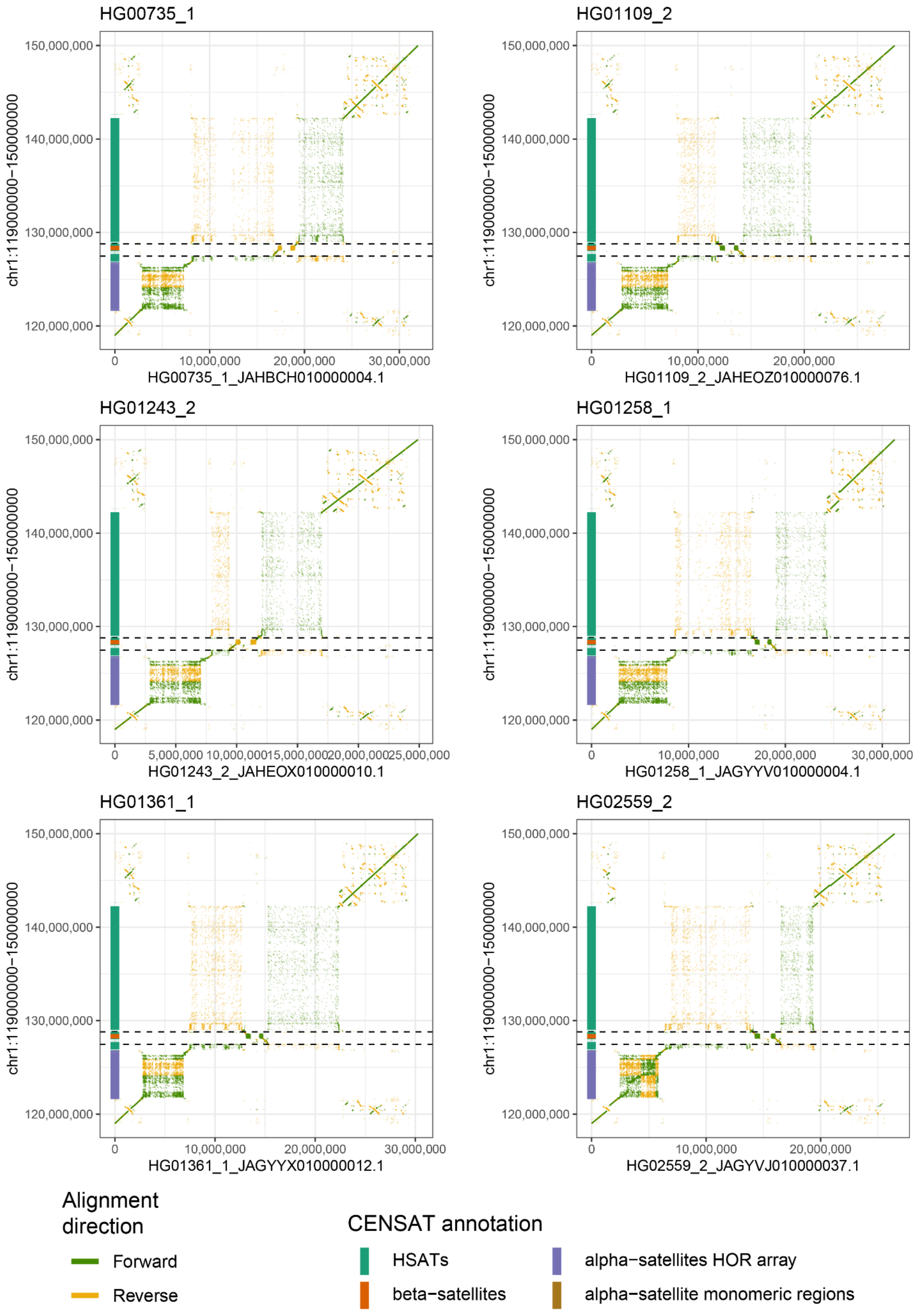

Figure S8: Complete assemblies of chromosome 1 centromeric region.
Dotplots show the alignment directionality (yellow - reverse, green - forward) between complete assemblies of chromosome 1 centromere (x-axis) against T2T-CHM13 reference (y-axis). T2T-CHM13 centromere annotation is shown as colored boxes on the y-axis.

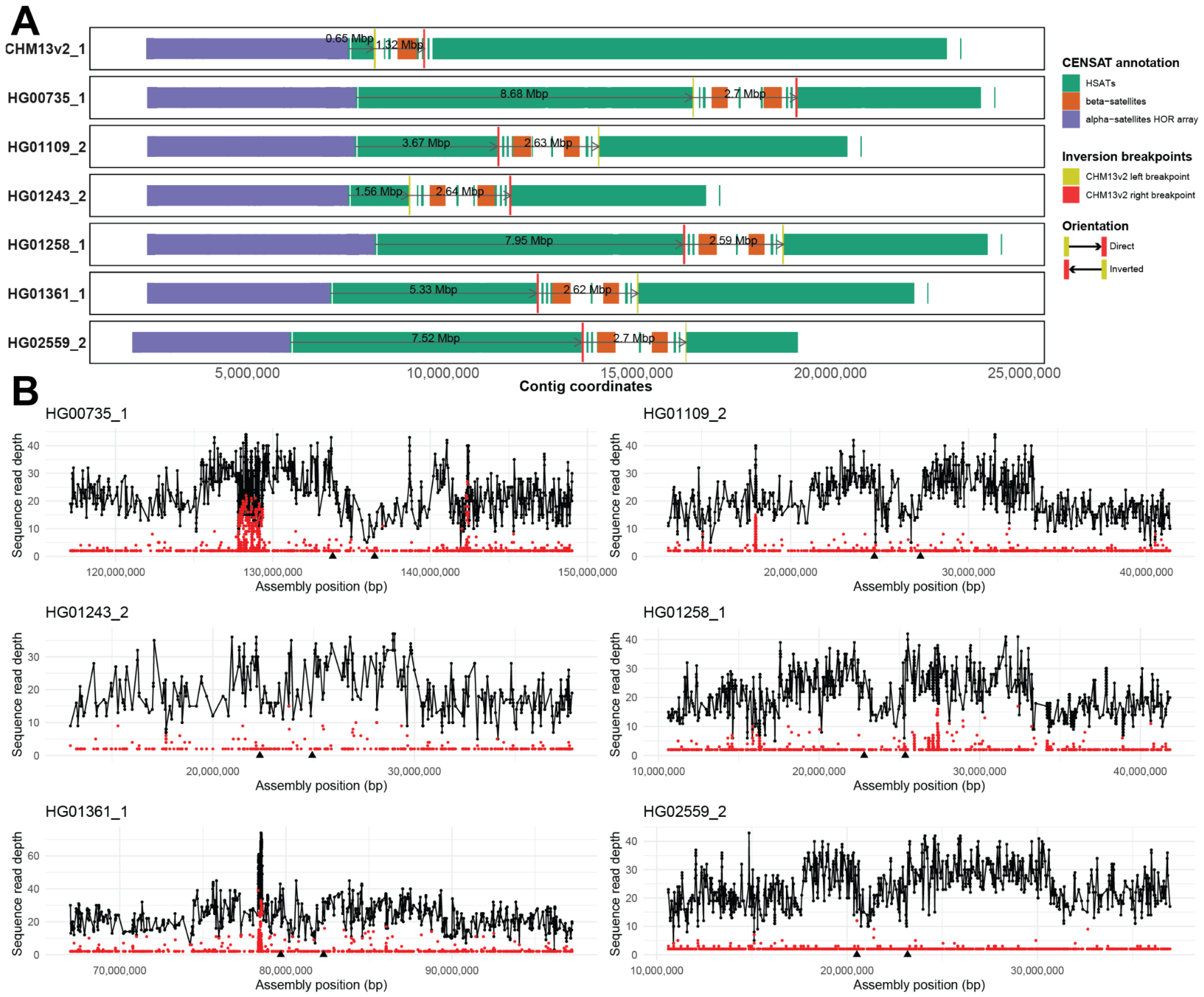

Figure S9: Relative position of alpha satellite array and novel pericentromeric inversion on chromosome 1.

A) RepeatMasker annotation of centromeric repeats for six complete assemblies of chromosome 1 centromere along with T2T-CHM13 reference (CHM13v2_1). We highlight alpha satellites (purple), beta satellites (orange) and human satellites (green). Mapped left (yellow) and right (red) inversion breakpoints are highlighted as vertical bars (**Methods**). Distances between boundaries of alpha satellite repeats and left-most inversion breakpoints are shown as an arrow with the distance in Mbp. Similarly, we show the distance between left and right inversion breakpoints. **B**) NucFreq validation of assembled centromeric regions presented in A). Black dots show read depth for the most common base at a given position while red dots show the second most common base. Regions where we observe high depth of the second most abundant base (red) are likely assembly collapses. Predicted inversion breakpoints are marked as black arrowheads at the bottom of each plot.

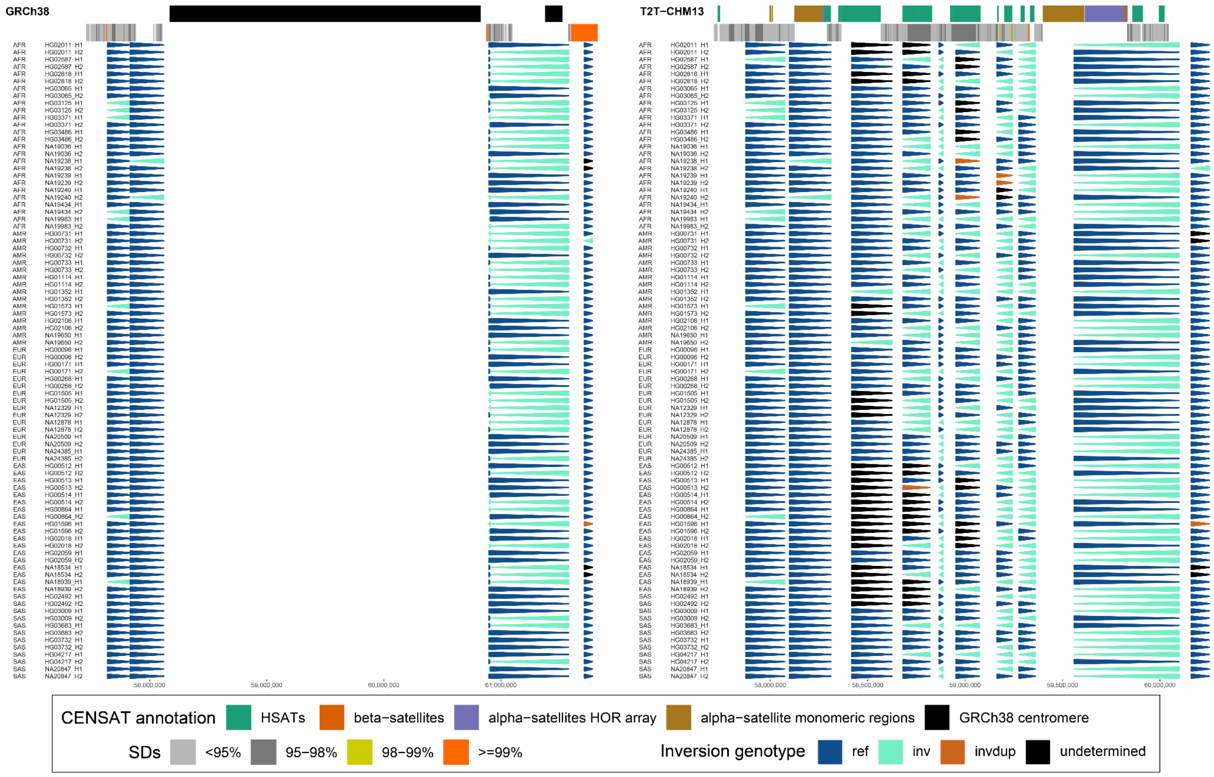

Figure S10: Inversion phasing at pericentromeric region of chromosome 7.
An arrowhead plot showing the inverted status of each defined region reported as colored arrowheads (dark blue - direct, bright blue - inverted, see the legend) for corresponding regions with respect to GRCh38 (left) and T2T-CHM13 (right). Gray rectangle in the middle highlights the positions of chromosome 7 centromere in GRCh38.

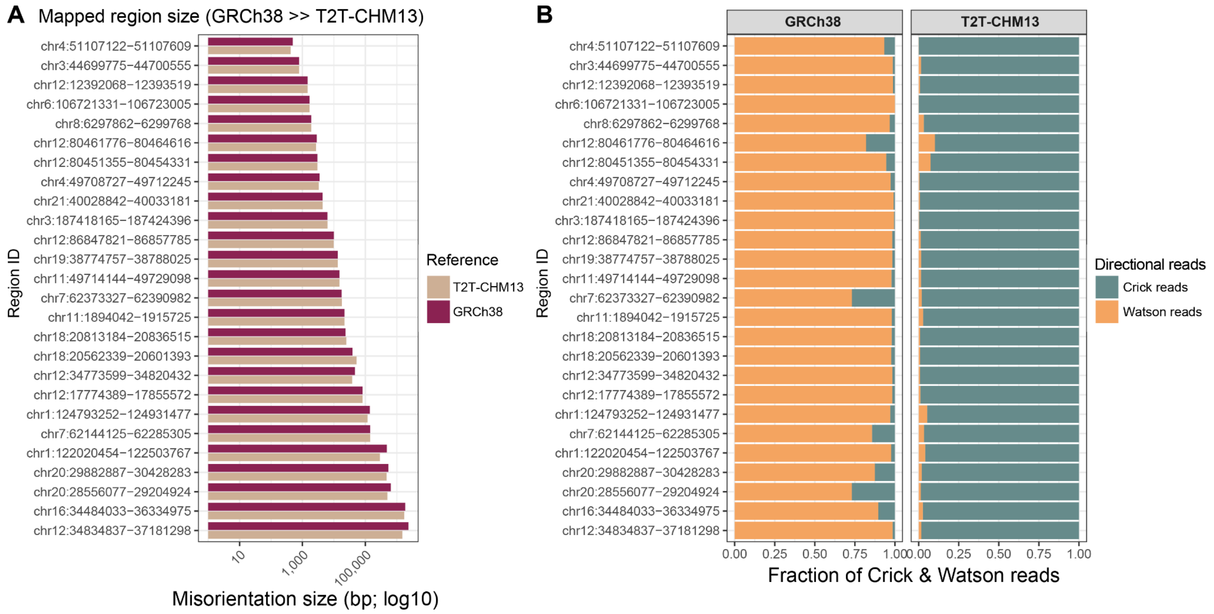

Figure S11: Evaluation of putative misorients in GRCh38 with respect to T2T-CHM13.
A) Distribution of region sizes of 28 putative misorients in GRCh38 (green) and their respective sizes after mapping onto the T2T-CHM13 reference genome (**Methods**). **B**) Fraction of Watson (minus; orange; wReads) and Crick (plus; teal; cReads) reads mapped to each region separately for reads mapped to GRCh38 and T2T-CHM13 reference genomes. Read counts are concatenated across all unrelated individuals (n=41) reported in this study.

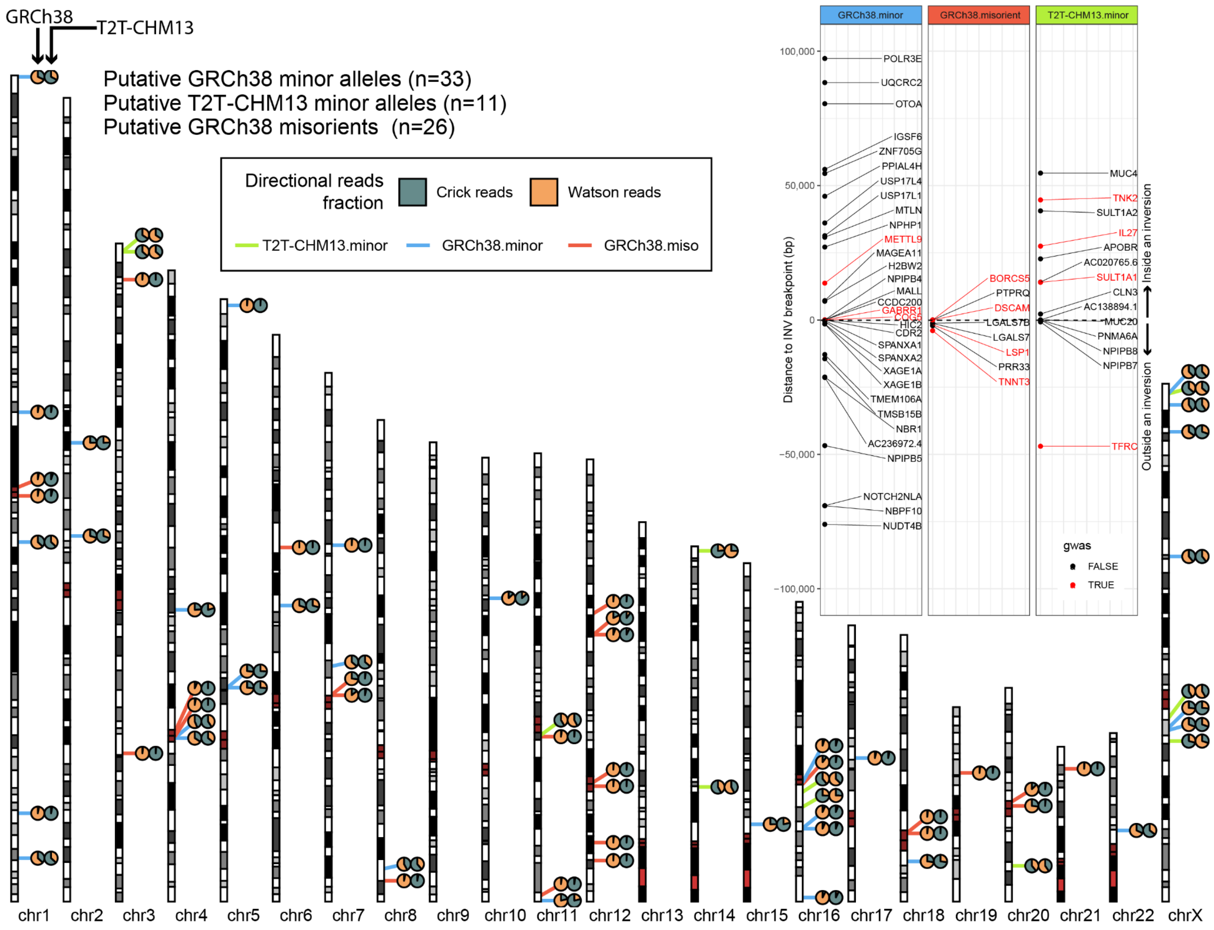

Figure S12: Evaluation of inversion differences between GRCh38 and T2T-CHM13 references. A GRCh38 ideogram shows the fraction of Watson (orange; minus) and Crick (teal; plus) reads aligned to both GRCh38 (left side pie) and T2T-CHM13 (right side pie) references for a selected number of regions. Strand-seq read counts are summarized across all unrelated individuals (n=41) from this study. Positions of putative minor alleles in GRCh38 (n=33, blue lines) reference with respect to T2T-CHM13 are highlighted by blue lines. Putative misorientations in GRCh38 (n=26) evaluated with respect to T2T-CHM13 are highlighted by red lines. Putative minor alleles in T2T-CHM13 (n=11) predicted with respect to GRCh38 are highlighted by green lines. **Inset:** Shows positions of protein-coding genes that reside within 100 kbp distance from GRCh38 misorientation (n=8), GRCh38 minor alleles (n=37) or T2T-CHM13 minor alleles (n=14). Gene names colored in red have been previously reported as part of genome-wide association studies (GWAS).

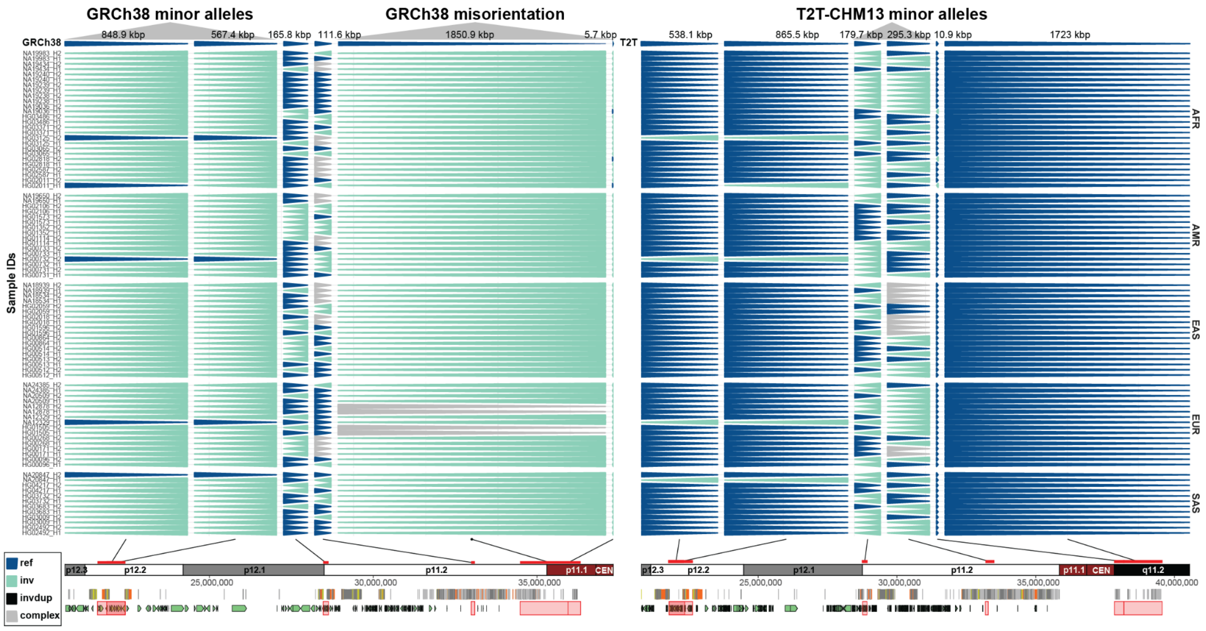

Figure S13: Examples of minor and misoriented alleles at chromosome 16.

A "backgammon" plot for a 20 Mbp region at chromosome 16p region depicting changes in the representation of major alleles as inverted (light blue) and direct orientation (dark blue) based on phased inversion genotypes reported with respect to GRCh38 and T2T-CHM13 reference genomes. In most cases, GRCh38 was either erroneous or represented the minor allele. Each horizontal set of arrowheads represents a single haplotype from diverse human populations (AFR - African, AMR - American, EAS - East Asian, EUR - European, and SAS - South Asian).

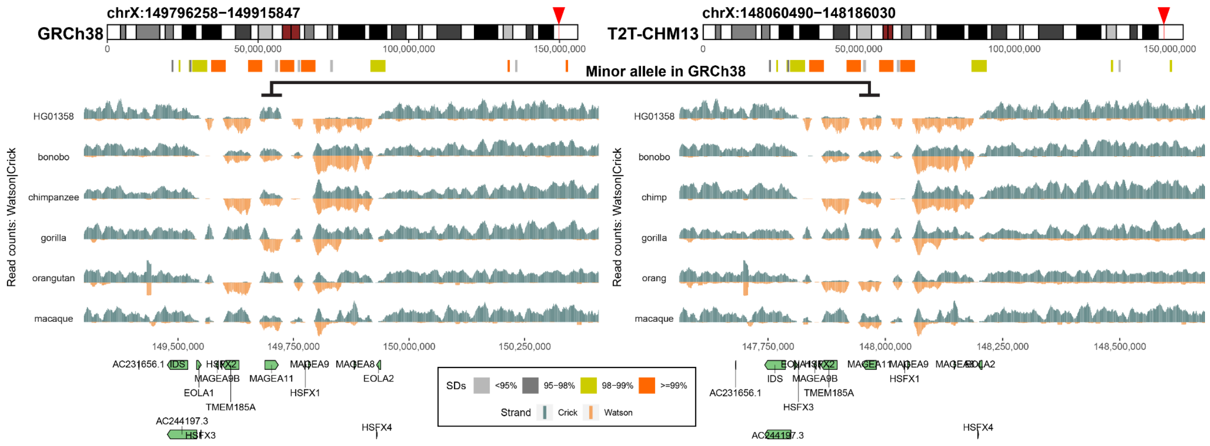

Figure S14: Structural differences at Xq28 between GRCh38 and T2T-CHM13.

Strand-seq read-coverage profiles over the Xq28 region summarized as binned (bin size: 10 kbp step size: 1 kbp) read counts represented as bars above (teal; Crick read counts) and below (orange; Watson read counts) the midline. Equal coverage of Watson and Crick counts represents a heterozygous inversion as only one homologue is inverted with respect to the reference while reads aligned only in Watson orientation represent a homozygous inversion. There is a novel inversion in sample HG01358 with respect to T2T-CHM13. A horizontal line shows a region where there is a minor allele in GRCh38.

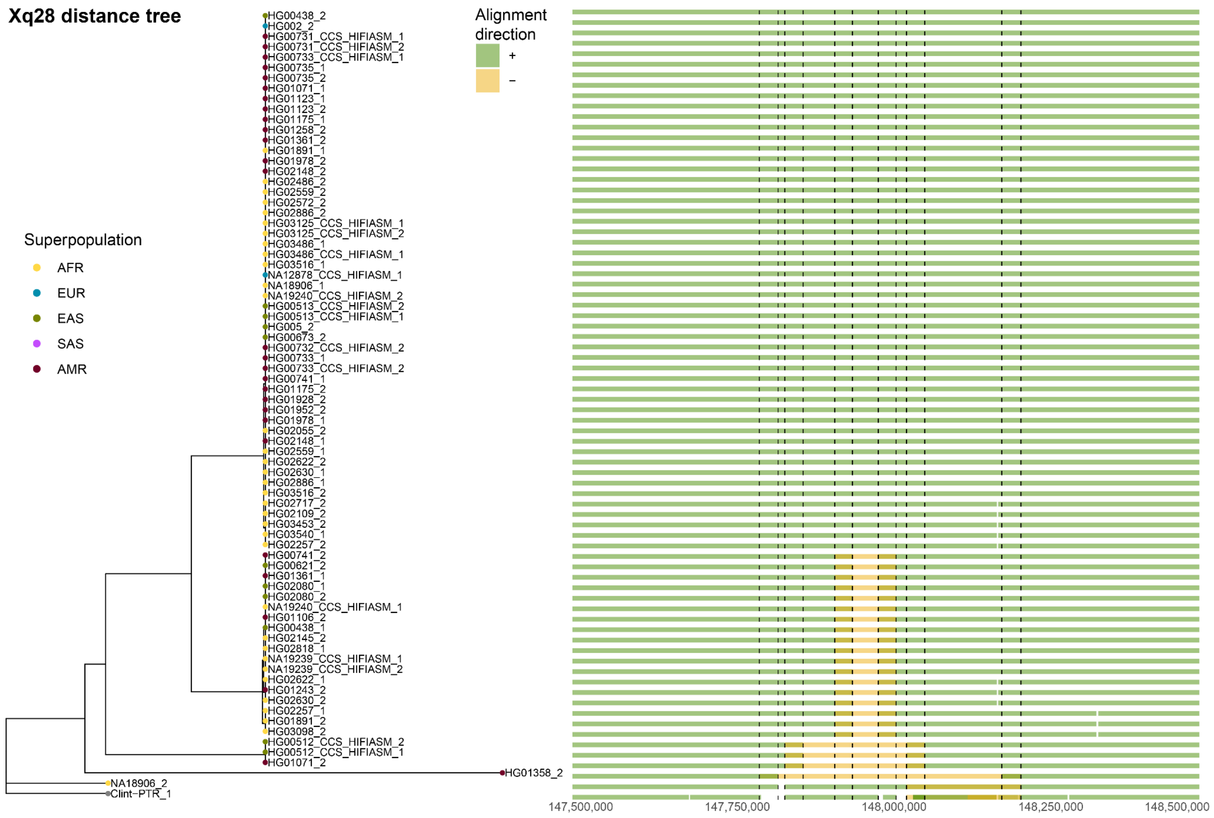

Figure S15: Diverse structural haplotypes at the Xq28 region.

Left: An UPGMA tree grouping complete assemblies (n=76) of the Xq28 region into structurally similar groups based on their alignment to T2T-CHM13 (**Methods**). Superpopulation of origin for each sample is marked by colored dots. Right: Visualization of alignment directionality (plus - green, minus - orange) of each assembly with respect to T2T-CHM13. Positions of SD blocks in the Xq28 region are highlighted by vertical dotted lines. Each alignment is plotted with 0.5 level of transparency such that overlapping alignments are visible as boxes with a darker color or a mixed green and orange color.

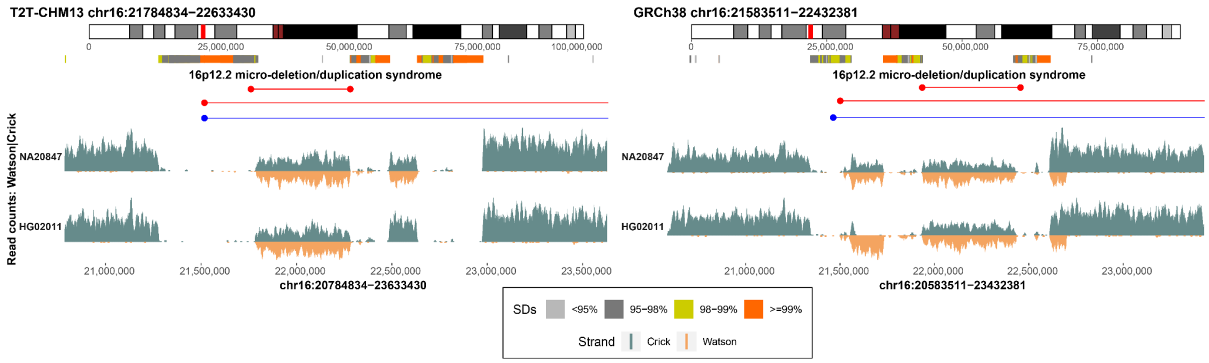

Figure S16: Structural differences at 16p12.2 between GRCh38 and T2T-CHM13.

Structural differences in the 16p12.2 region are visualized as read-coverage profiles of Strand-seq reads mapped separately to GRCh38 (right plot) and T2T-CHM13 (left plot) references. Strand-seq reads are summarized as binned (bin size: 10 kbp, step size: 1 kbp) read counts represented as bars above (teal; Crick read counts) and below (orange; Watson read counts) the midline. The region with roughly equal coverage of Watson and Crick counts represents a heterozygous inversion as only one homologue is inverted with respect to the reference while the region with reads aligned only in Watson orientation represents a homozygous inversion. Each inverted region is highlighted on a chromosome-specific ideogram by a red rectangle.

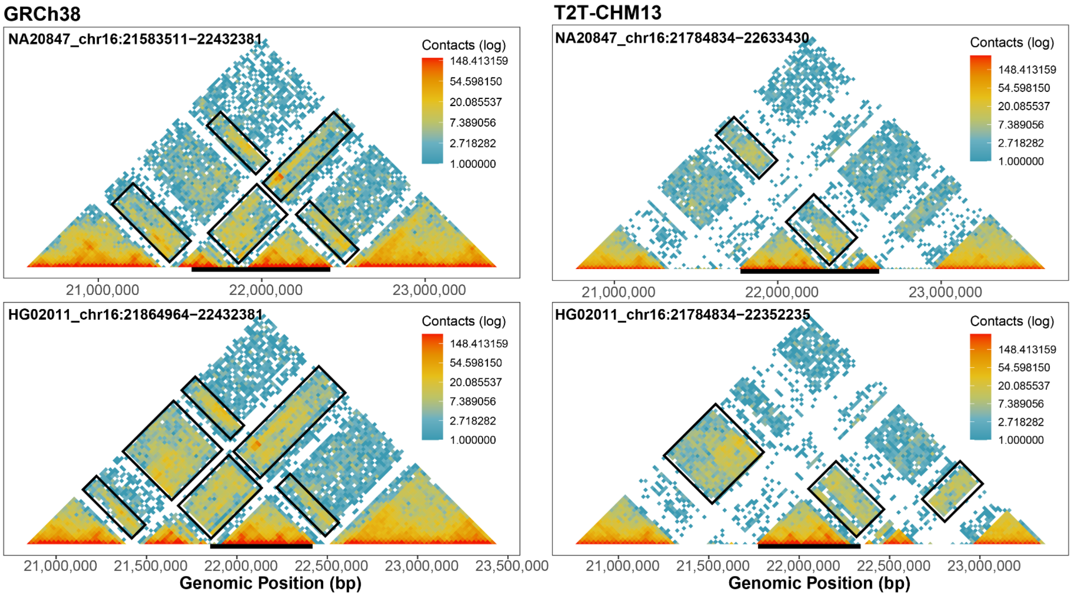

Figure S17: Topological differences at 16p12.2 between GRCh38 and T2T-CHM13.
Contact matrices deduced based on Hi-C data mapped to GRCh38 and T2T-CHM13 reference are shown on the left and right side, respectively. We present contact matrices constructed for two samples (HG02011 and GM20847) with short and long versions of the inversion over the selected chromosome 16 region (black bar at the bottom). Intensity of contacts between proximal regions of the genome is represented by a heatmap colors from low level of contacts (blue) to high level of contacts (red). Regions with different levels of contact between two matrices are highlighted by black rectangles.

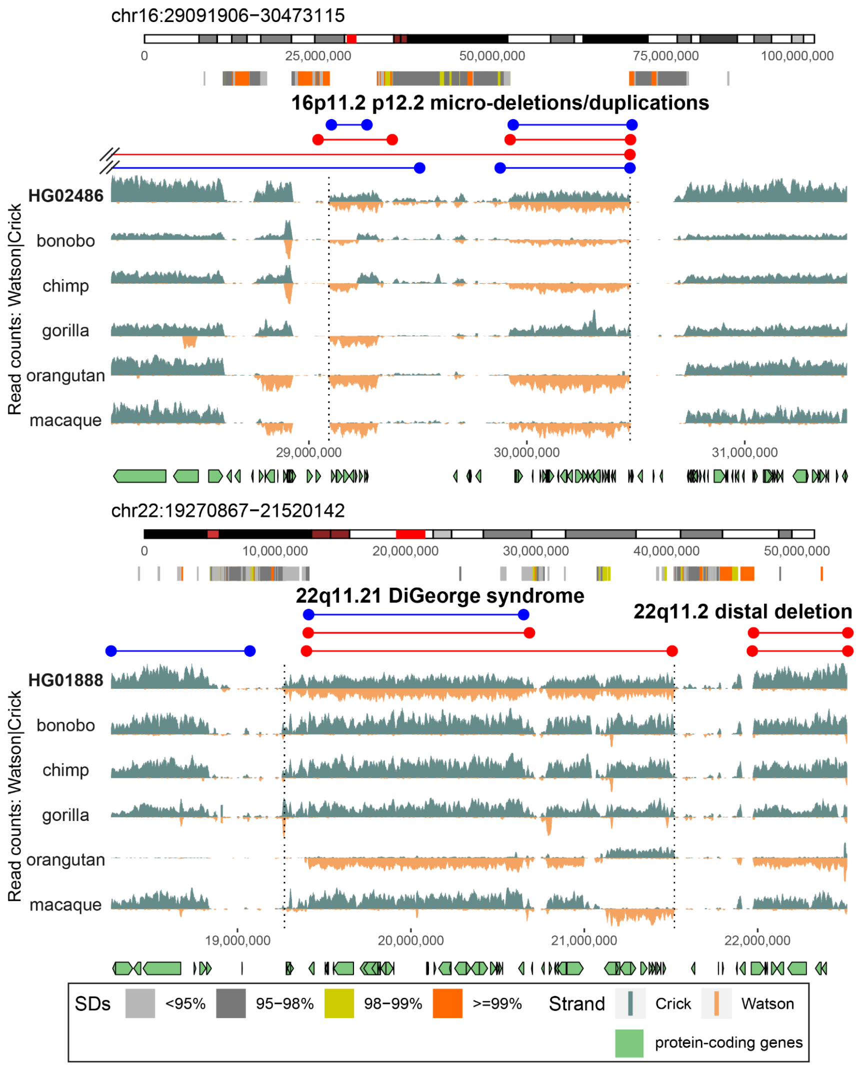

Figure S18: Rare inversions at disease relevant loci.

Two disease-associated regions mapping to chromosomes 16p11.2 and 22q11.21 are depicted within chromosome-specific ideograms (red rectangle) with a zoom into the region flanked by segmental duplications (colored horizontal bars) and pathogenic duplication and deletion breakpoints highlighted in blue and red horizontal lines, respectively. Strand-seq data highlights rare heterozygous inversions (see **Fig. 1C** for detailed description) discovered in a human sample with respect to the status in different nonhuman primate species. Homozygous inversions are orange while homozygous teal represent homozygous direct orientations.

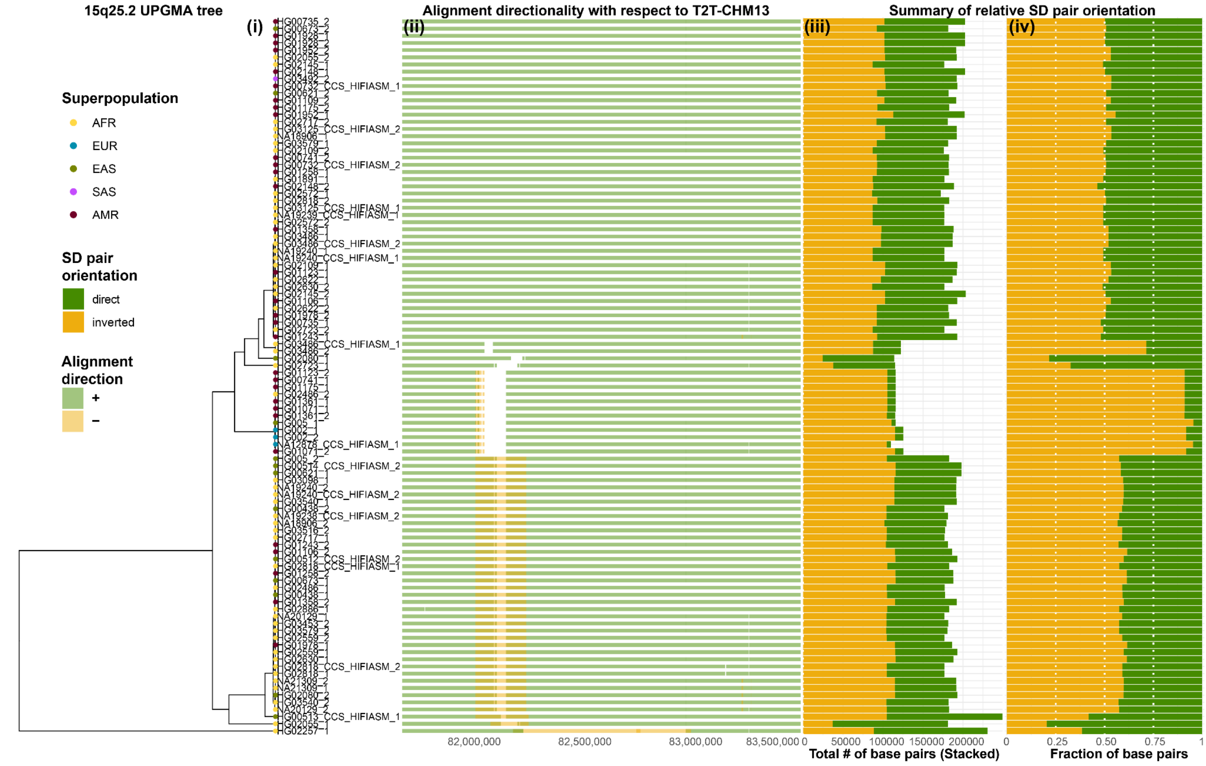

Figure S19: Diverse structural haplotypes at 15q25.2 region.

From left to right: (i) An UPGMA tree grouping complete assemblies (n=101) of 15q25.2 region into structurally similar groups based on their alignment to T2T-CHM13 (**Methods**). Superpopulation of origin for each sample is marked by colored dots. (ii) Visualization of alignment directionality (plus - green, minus - orange) of each assembly with respect to T2T-CHM13. (iii) Summary of the total number of base pairs for direct and reverse orientated SD pairs. (iv) Summary of the fraction of base pairs for direct and reverse orientated SD pairs.

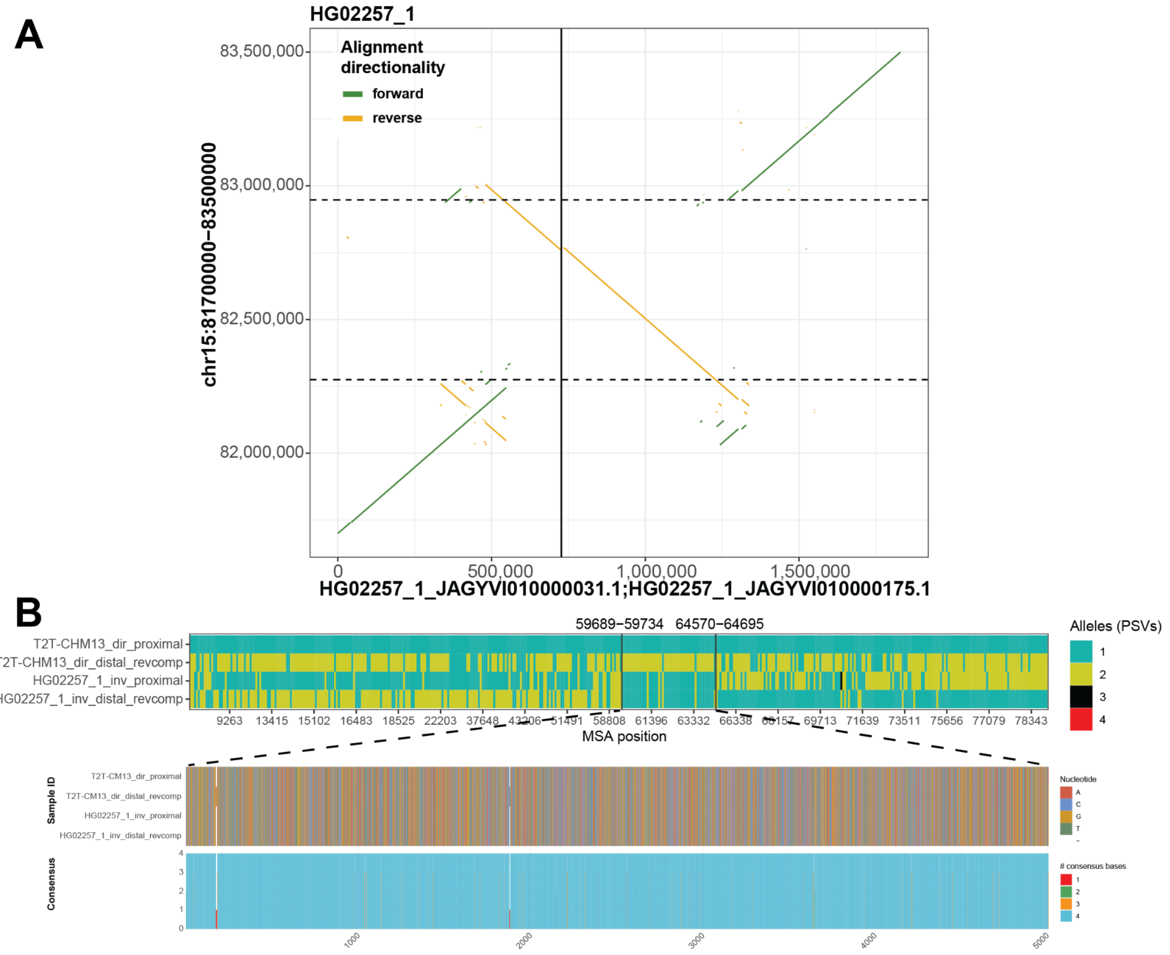

Figure S20: Assembled inversion breakpoints at 15q25.2 and inversion breakpoint mapping.
A) Dotplot of the alignment directionality (yellow - reverse, green - forward) for the HG02257 assembly of 15q25.2 region (x-axis) against the T2T-CHM13 reference (y-axis). The reported inversion in T2T-CHM13 coordinates is highlighted by horizontal dashed lines. The position where one contig ends and another starts is marked by a solid vertical line. **B**) Visualization of multiple sequence alignment (MSA) between inversion flanking SDs from direct (T2T-CHM13) and inverted (HG02257) haplotypes. Only paralog-specific variants (PSVs) from the proximal (bright green) and distal (dark yellow) SDs are colored separately. Gaps in the MSA are colored white and alleles not present in the proximal and distal SDs are shown in black and red, respectively. Vertical solid lines depict detected change points, with numbers showing the change point position within flanking SDs. We predict that the inversion breakpoints lie between the 59,689 and 64,695 bp of flanking SDs. Below we zoom into a ~5 kbp wide breakpoint region of high homology shown by almost perfect consensus across inversion flanking SDs.

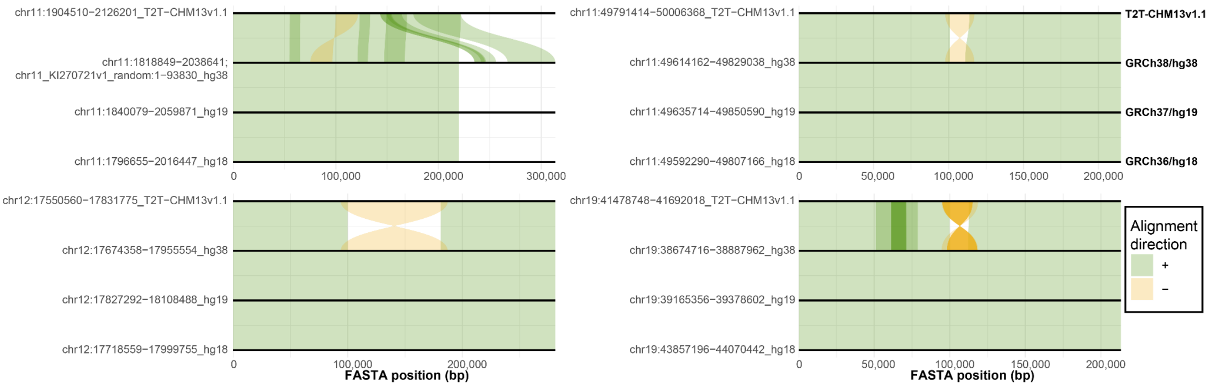

Figure S21: Example of long-lasting misorientation errors in previous human genome references.

Here, we show a comparison of the FASTA sequences extracted from four misoriented regions (**Additional file 2: Table S4**) across four versions of human genome reference assemblies (from top to bottom: T2T-CHM13 v1.1, GRCh38, GRCh37 and GRCh36). Alignment directionality is highlighted by direct ('+', green) and reverse ('-', orange) oriented flows between pairs of FASTA sequences.

SUPPLEMENTARY NOTES:

Rationale for focusing on balanced inversions:

We focused on balanced inversions primarily because balanced inversions are more amenable to genotyping using Strand-seq data and phased genome assemblies and, as such, we can estimate more accurate inversion population frequencies. In contrast, regions classified as inverted duplications are only telling us that a given region is duplicated and inverted in a given genome with respect to the reference. However, this is not conclusive as to where this duplicated region is positioned in the genome in question. Similarly, complex events are difficult to genotype using Strand-seq due to the insufficient mappability of short Strand-seq reads and, thus, we can only tell that there might be a change in orientation in a given region but the full extent of complexity is difficult to resolve.

Consortia:

Human Genome Structural Variation Consortium (HGSVC):

HGSVC co-chairs:
Charles Lee, Evan E. Eichler, Jan O. Korbel, Tobias Marschall

European Molecular Biology Laboratory (EMBL), Genome Biology Unit, Meyerhofstr. 1, 69117 Heidelberg, Germany
Jan O. Korbel, Bernardo Rodriguez-Martin, Tobias Rausch, Marc Jan Bonder, Wolfram Höps, Ashley D. Sanders, Benjamin Raeder, Patrick Hasenfeld, Oliver Stegle

Heinrich Heine University, Medical Faculty, Institute for Medical Biometry and Bioinformatics, Moorenstr. 20, 40225 Düsseldorf, Germany
Tobias Marschall, Peter Ebert, Jana Ebler, Hufsah Ashraf, Rebecca Serra Mari, Maryam Ghareghani

Department of Genome Sciences, University of Washington School of Medicine, Seattle, WA 98195, USA
Evan E. Eichler, David Porubsky, PingHsun Hsieh, Katherine M. Munson, William T. Harvey, Alexandra P. Lewis

The Jackson Laboratory for Genomic Medicine, Farmington, CT 06032, USA
Charles Lee, Christine Beck, Peter A. Audano, Qihui Zhu, Feyza Yilmaz, Pille Hallast

Department of Computational Medicine & Bioinformatics, University of Michigan, Ann Arbor, MI 48109, USA
Ryan E. Mills, Weichen Zhou

Institute for Genome Sciences, University of Maryland School of Medicine, Baltimore, MD 21201, USA
Scott E. Devine, Nelson T. Chuang, Luke J. Tallon

Center for Genomic Medicine, Massachusetts General Hospital, Department of Neurology, Harvard Medical School, Boston, MA 02114, USA
Michael E. Talkowski, Xuefang Zhao, Harrison Brand

Program in Computational Biology and Bioinformatics, Yale University, New Haven, CT 06520, USA

Mark B. Gerstein, Sushant Kumar

Xi'an Jiaotong University, Xi'an, Shaanxi, 710049, China
Kai Ye, Jiadong Lin, Xiaofei Yang

Department of Quantitative and Computational Biology, University of Southern California, Los Angeles, CA 90089, USA
Mark J.P. Chaisson, Jingwen Ren, Tsung-Yu Lu

Department of Computer & Information Sciences, Temple University, Philadelphia, PA 19122, USA
Xinghua Shi, Chong Li, Sky Gao, Bin Li, Chen Song

Department of Genetics, Yale School of Medicine, New Haven, CT 06510, USA
Ira M. Hall

Department of Genetics and Informatics Institute, School of Medicine, University of Alabama at Birmingham, Birmingham, AL 35294, USA
Zechen Chong, Yu Chen

European Molecular Biology Laboratory, European Bioinformatics Institute, Wellcome Genome Campus, Hinxton, Cambridge, CB10 1SD, United Kingdom
Sarah Hunt, Susan Fairley, Paul Flicek

New York Genome Center, New York, NY 10013, USA
Michael C. Zody, Wayne E. Clarke, Anna O. Basile, Marta Byrska-Bishop, André Corvelo, Uday S. Evani

Washington University, St. Louis, MO 63108, USA
Allison A. Regier, Haley J. Abel

University of Chicago, Chicago, IL 60637, USA
Yang I. Li, Zepeng Mu

Department of Bioinformatics and Computational Biology, The University of Texas MD Anderson Cancer Center, Houston, TX 77030, USA
Ken Chen

Genomes and Disease, Centre for Research in Molecular Medicine and Chronic Diseases (CIMUS), Universidade de Santiago de Compostela, Santiago de Compostela, Spain
Martin Santamarina, Jose M.C. Tubio

Bionano Genomics, San Diego, CA 92121, USA
Alex R. Hastie

Pacific Biosciences of California, Inc., Menlo Park, CA 94025, USA
Aaron M. Wenger

Human Pangenome Reference Consortium (HPRC)

Haley J. Abel[1], Lucinda L. Antonacci-Fulton[2], Mobin Asri[3], Gunjan Baid[4], Carl A. Baker[5], Anastasiya Belyaeva[4], Konstantinos Billis[6], Guillaume Bourque[7,8,9], Silvia Buonaiuto[10], Andrew Carroll[4], Mark J. P. Chaisson[11], Pi-Chuan Chang[4], Xian H. Chang[3], Haoyu Cheng[12,13], Justin Chu[12], Sarah Cody[2], Vincenza Colonna[10,14], Daniel E. Cook[4], Robert M. Cook-Deegan[15], Omar E. Cornejo[16], Mark Diekhans[3], Daniel Doerr[17,18], Peter Ebert[17,19,18], Jana Ebler[17,18], Evan E. Eichler[5,20], Jordan M. Eizenga[3], Susan Fairley[6], Olivier Fedrigo[21], Adam L. Felsenfeld[22], Xiaowen Feng[12,13], Christian Fischer[14], Paul Flicek[6], Giulio Formenti[21], Adam Frankish[6], Robert S. Fulton[2,23], Yan Gao[24], Shilpa Garg[25], Erik Garrison[14,+], Nanibaa' A. Garrison[26,27,28], Carlos Garcia Giron[6], Richard E. Green[29,30], Cristian Groza[31], Andrea Guarracino[32,14], Leanne Haggerty[6], Ira M. Hall[33,34], William T. Harvey[5], Marina Haukness[3], David Haussler[3,20], Simon Heumos[35,36], Glenn Hickey[3], Kendra Hoekzema[5], Thibaut Hourlier[6], Kerstin Howe[37], Miten Jain[38], Erich D. Jarvis[21,20,39], Hanlee P. Ji[40], Eimear E. Kenny[41], Barbara A. Koenig[42], Alexey Kolesnikov[4], Jan O. Korbel[6,43], Jennifer Kordosky[5], Sergey Koren[44], HoJoon Lee[40], Alexandra P. Lewis[5], Heng Li[12,13], Wen-Wei Liao[33,34,45], Shuangjia Lu[33], Tsung-Yu Lu[11], Julian K. Lucas[3], Hugo Magalhães[17,18], Santiago Marco-Sola[46,47], Pierre Marijon[17,18], Charles Markello[3], Tobias Marschall[17,18], Fergal J. Martin[6], Ann McCartney[44], Jennifer McDaniel[48], Karen H. Miga[3], Matthew W. Mitchell[49], Jean Monlong[3], Jacquelyn Mountcastle[21], Katherine M. Munson[5], Moses Njagi Mwaniki[50], Maria Nattestad[4], Adam M. Novak[3], Sergey Nurk[44], Hugh E. Olsen[3], Nathan D. Olson[48], Benedict Paten[3], Trevor Pesout[3], Adam M. Phillippy[44], Alice B. Popejoy[51], David Porubsky[5], Pjotr Prins[14], Daniela Puiu[52], Mikko Rautiainen[44], Allison A. Regier[2], Arang Rhie[44], Samuel Sacco[53], Ashley D. Sanders[54], Valerie A. Schneider[55], Baergen I. Schultz[22], Kishwar Shafin[4], Jonas A. Sibbesen[56], Jouni Sirén[3], Michael W. Smith[22], Heidi J. Sofia[22], Ahmad N. Abou Tayoun[57,58], Françoise Thibaud-Nissen[55], Chad Tomlinson[2], Francesca Floriana Tricomi[6], Flavia Villani[14], Mitchell R. Vollger[5,59], Justin Wagner[48], Brian Walenz[44], Ting Wang[2,23], Jonathan M. D. Wood[37], Aleksey V. Zimin[52,60], Justin M. Zook[48]

1 Division of Oncology, Department of Internal Medicine, Washington University School of Medicine, St. Louis, MO 63110, USA

2 McDonnell Genome Institute, Washington University School of Medicine, St. Louis, MO 63108, USA

3 UC Santa Cruz Genomics Institute, University of California, Santa Cruz, Santa Cruz, CA 95064, USA

4 Google LLC, Mountain View, CA 94043, USA

5 Department of Genome Sciences, University of Washington School of Medicine, Seattle, WA 98195, USA

6 European Molecular Biology Laboratory, European Bioinformatics Institute, Wellcome Genome Campus, Hinxton, Cambridge CB10 1SD, UK

7 Department of Human Genetics, McGill University, Montreal, Québec H3A 0C7, Canada

8 Canadian Center for Computational Genomics, McGill University, Montreal, Québec H3A 0G1, Canada

9 Institute for the Advanced Study of Human Biology (WPI-ASHBi), Kyoto University, Kyoto 606-8501, Japan
10 Institute of Genetics and Biophysics, National Research Council, Naples 80111, Italy
11 Department of Quantitative and Computational Biology, University of Southern California, Los Angeles, CA 90089, USA
12 Department of Data Sciences, Dana-Farber Cancer Institute, Boston, MA 02215, USA
13 Department of Biomedical Informatics, Harvard Medical School, Boston, MA 02215, USA
14 Department of Genetics, Genomics and Informatics, University of Tennessee Health Science Center, Memphis, TN 38163, USA
15 Arizona State University, Barrett and O'Connor Washington Center, Washington DC 20006, USA
16 Department of Ecology and Evolutionary Biology, University of California, Santa Cruz, Santa Cruz, CA 95064, USA
17 Institute for Medical Biometry and Bioinformatics, Medical Faculty, Heinrich Heine University Düsseldorf, Düsseldorf 40225, Germany
18 Center for Digital Medicine, Heinrich Heine University Düsseldorf, Düsseldorf 40225, Germany
19 Core Unit Bioinformatics, Medical Faculty, Heinrich Heine University Düsseldorf, Düsseldorf 40225, Germany
20 Howard Hughes Medical Institute, Chevy Chase, MD 20815, USA
21 Vertebrate Genome Laboratory, The Rockefeller University, New York, NY 10065, USA
22 National Institutes of Health (NIH)–National Human Genome Research Institute, Bethesda, MD 20892, USA
23 Department of Genetics, Washington University School of Medicine, St. Louis, MO 63110, USA
24 Center for Computational and Genomic Medicine, The Children’s Hospital of Philadelphia, Philadelphia, PA 19104, USA
25 Novo Nordisk Foundation Center for Biosustainability, Technical University of Denmark, Copenhagen DK-2200, Denmark
26 Institute for Society and Genetics, College of Letters and Science, University of California, Los Angeles, Los Angeles, CA 90095, USA
27 Institute for Precision Health, David Geffen School of Medicine, University of California, Los Angeles, Los Angeles, CA 90095, USA
28 Division of General Internal Medicine and Health Services Research, David Geffen School of Medicine, University of California, Los Angeles, Los Angeles, CA 90095, USA
29 Department of Biomolecular Engineering, University of California, Santa Cruz, Santa Cruz, CA 95064, USA
30 Dovetail Genomics, Scotts Valley, CA 95066, USA
31 Quantitative Life Sciences, McGill University, Montreal, Québec H3A 0C7, Canada
32 Genomics Research Centre, Human Technopole, Milan 20157, Italy
33 Department of Genetics, Yale University School of Medicine, New Haven, CT 06510, USA
34 Center for Genomic Health, Yale University School of Medicine, New Haven, CT 06510, USA
35 Quantitative Biology Center (QBiC), University of Tübingen, Tübingen 72076, Germany

36 Biomedical Data Science, Department of Computer Science, University of Tübingen, Tübingen 72076, Germany
37 Tree of Life, Wellcome Sanger Institute, Hinxton, Cambridge CB10 1SA, UK
38 Northeastern University, Boston, MA 02115, USA
39 Laboratory of Neurogenetics of Language, The Rockefeller University, New York, NY 10065, USA
40 Division of Oncology, Department of Medicine, Stanford University School of Medicine, Stanford, CA, 94305, USA
41 Institute for Genomic Health, Icahn School of Medicine at Mount Sinai, New York, NY 10029, USA
42 Program in Bioethics and Institute for Human Genetics, University of California, San Francisco, San Francisco, CA, USA
43 European Molecular Biology Laboratory, Genome Biology Unit, Heidelberg 69117, Germany
44 Genome Informatics Section, Computational and Statistical Genomics Branch, National Human Genome Research Institute, National Institutes of Health, Bethesda, MD 20892, USA
45 Division of Biology and Biomedical Sciences, Washington University School of Medicine, St. Louis, MO 63110, USA
46 Computer Sciences Department, Barcelona Supercomputing Center, Barcelona, Spain
47 Departament d'Arquitectura de Computadors i Sistemes Operatius, Universitat Autònoma de Barcelona, Barcelona, Spain
48 Material Measurement Laboratory, National Institute of Standards and Technology, Gaithersburg, MD 20877, USA
49 Coriell Institute for Medical Research, Camden, NJ 08103, USA
50 Department of Computer Science, University of Pisa, Pisa 56127, Italy
51 Department of Public Health Sciences, University of California, Davis, Davis, CA 95616, USA
52 Department of Biomedical Engineering, Johns Hopkins University, Baltimore 21218, MD, USA
53 Department of Ecology and Evolutionary Biology, University of California, Santa Cruz, Santa Cruz, CA 95064, USA
54 Berlin Institute for Medical Systems Biology, Max Delbrück Center for Molecular Medicine in the Helmholtz Association, Berlin 10115, Germany
55 National Center for Biotechnology Information, National Library of Medicine, National Institutes of Health, Bethesda, MD 20894, USA
56 Center for Health Data Science, University of Copenhagen, Copenhagen 2200, Denmark
57 Al Jalila Genomics Center of Excellence, Al Jalila Children's Specialty Hospital, Dubai, UAE
58 Center for Genomic Discovery, Mohammed Bin Rashid University of Medicine and Health Sciences, Dubai, UAE
59 Division of Medical Genetics, University of Washington School of Medicine, Seattle, WA 98195, USA
60 Center for Computational Biology, Johns Hopkins University, Baltimore, MD 21218, USA
