# Supplementary material for: Inversion polymorphism in a complete human genome assembly
Source: Genome Biol. 2023 Apr 30;24:100. doi: 10.1186/s13059-023-02919-8 (PMC10150506; doi:10.1186/s13059-023-02919-8)
Supplement: Supplementary file 3 — Additional file 3. Review history. [file 13059_2023_2919_MOESM3_ESM.docx]

Review History

**First round of review**

**Reviewer 1**

**Were you able to assess all statistics in the manuscript, including the appropriateness of statistical tests used?** Yes, and I have assessed the statistics in my report.

**Comments to author:**

The work presented in Porubsky et al. is of high value to the research community and is well done, overall. There are several items that need to be addressed before publication. I mark them as "minor" only because they are all easily addressed, and not because they lack import.

Unless I have misunderstood the paper, one of the biggest issues to address is that the paper reads very biased towards Strand-seq and ignores other approaches that appear to have also been helpful in the results. Given the conflicts of interest from several authors related to Strand-seq, they should be especially careful to avoid bias in their writing.

I also feel like the authors could provide significantly more summary information and stats regarding their findings, and can clarify throughout the paper exactly which numbers are being compared.

I may have overlooked it, but I do not see any indication that their scripts, etc. to reproduce the results are available. For reproducibility, this is a requirement, in my view.

To the authors: Excellent and exciting work. My comments are sincerely meant to be helpful to improve the paper, especially for the readers. I hope you find them helpful.

Sincerely,

Mark T. W. Ebbert

Minor:

1. The following statement likely needs to be expounded and qualified: "While various approaches have been developed over the years, the Strand-seq platform among the most sensitive".

a. Potentially misleading and biased: While Strand-seq may be "among" the most sensitive, I feel like this statement is misleading and should be qualified. Specifically, it only mentions one of the approaches used in this paper (i.e., Strand-seq) and ignores the other methods used in this paper's primary results (i.e., standard long-read sequencing and optical DNA mapping)! Specifically, the second sentence in the results states the following: "…and combined it with both Bionano Genomics and assembly-based approaches to detect inversions". If (as scientists) we want to educate our readers without bias, we should be careful to educate them fully—especially in the introduction to the paper. If this paper were only using Strand-seq, I could understand why the introduction would only that Strand-seq is "among" the most sensitive, but the work clearly includes other approaches in the primary results, suggesting these other technologies added valuable information. As the authors acknowledge, there is a clear conflict of interest related to Strand-seq. Please give credit where credit is due.

b. Additionally, I believe sensitivity for each platform varies by structural variant size (e.g., Bionano often does better on the larger variants than standard long-read sequencing approaches, while long-read seq typically excels with smaller variants). I quickly read through one of the cited papers (Porubsky et al. 2022, Cell) to see if it supports the broad claim above. I was surprised to see that this paper makes an even broader statement, which may have been even more misleading. I could be wrong, but I couldn't find sufficient data in the Cell paper to support the broad claim. Specifically, the Cell paper acknowledges that standard long-read sequencing increases sensitivity for "events smaller than 100kbp", but then broadly states that "Bionano technology is least sensitive…", citing Figure 1D. Unless I misinterpreted Figure 1D in Porubsky et al. 2022 (Cell), I do not see any stratification by structural variant size to indicate that Bionano is "least sensitive" across the entire spectrum of variant sizes. Nor did I see additional figures or stats supporting or clarifying this statement, but I may have overlooked it. I did not read the other cited papers, but it would helpful if the authors could either clarify/qualify throughout the paper, or persuade me that I am mistaken.

2. Same issue as above for the following statement in introduction: "We specifically focused on the analysis of Strand-seq data generated from previous…"

3. The following statement needs to be clarified either for me, or the readers (or both). i.e., why do you only focus on those variants? Why are you excluding the rest? "For the remainder of this study, we focus exclusively on analysis of 296 balanced inversions, 56 inverted duplications, and 21 complex events…" Those variants are at least as interesting as the balanced inversions.

4. Please forgive me if I'm misunderstanding, but the following statement in the results is a bit ambiguous. It may also be misleading depending on exactly which numbers are being compared. Please include the equation and percentage next to each stat for clarity: "Strand-seq detected the greatest proportion of inverted base pairs exclusively detecting 82 inversions with median size ~144kbp and corresponding to ~80Mbp of sequence."

a. As I understand, Strand-seq detected 82 of the 296 (82/296 = 28%) balanced inversions mentioned above? But those 82 account for ~80Mbp of the 82.8Mbp (~97%?) referenced above? Or is this 80Mbp of "all" inverted bases? Part of the reason this is unclear to me is that a preceding statement "clarifies" that the stats in that sentence are only "(considering balanced inversions only)". Incidentally, the results do not report the total number of *all* inverted bases. More summary statistics across all the variants would be extremely informative for the readers.

b. The reason this feels misleading is that it ignores that ~72% of the balanced inversions were presumably identified with the help of other approaches. Even if those 72% of events only account for ~3% of the nucleotides, they may be equally, or even more important. For example, maybe those 3% are directly affecting gene bodies or haplotypes of gene bodies, where perhaps the 97% of nucleotides are not. It would be great to see those stats, too. Of course, even if the 97% are not enriched for gene bodies, they may still be super important, but please provide more information!

5. For figure 2A, the circles for "Inverted Allele Count" in the legend are not to-scale with what is presented in the figure itself, which makes the interpretation more ambiguous.

6. Would be helpful to indicate chromosome and region withing figure 2B. I realize the text specifies, but it's not clear in the figure itself.

7. The methods state authors used LiftOver, but lift overs have a lot of inaccuracies. The authors state that all balanced inversions successfully lifted over. Please clarify how "success" is defined. Were these also manually curated?

8. To properly educate the readers, when providing specific tool parameters in the methods, please indicate their meaning, purpose, and why the authors deviated from default.

9. Unless I overlooked it, the authors do not provide their exact scripts/pipeline to reproduce their results. This should ideally also include a Singularity container, or related environment.

**Reviewer 2**

**Were you able to assess all statistics in the manuscript, including the appropriateness of statistical tests used?** No, I do not feel adequately qualified to assess the statistics.

**Comments to author:**

This Referee did not have any concerns and recommended acceptance of the manuscript.

**Authors Response**

**Reviewer #1:**

The work presented in Porubsky et al. is of high value to the research community and is well done, overall. There are several items that need to be addressed before publication. I mark them as "minor" only because they are all easily addressed, and not because they lack importance.

Unless I have misunderstood the paper, one of the biggest issues to address is that the paper reads very biased towards Strand-seq and ignores other approaches that appear to have also been helpful in the results. Given the conflicts of interest from several authors related to Strand-seq, they should be especially careful to avoid bias in their writing.

I also feel like the authors could provide significantly more summary information and stats regarding their findings, and can clarify throughout the paper exactly which numbers are being compared.

I may have overlooked it, but I do not see any indication that their scripts, etc. to reproduce the results are available. For reproducibility, this is a requirement, in my view.

To the authors: Excellent and exciting work. My comments are sincerely meant to be helpful to improve the paper, especially for the readers. I hope you find them helpful.

Sincerely,

Mark T. W. Ebbert

We thank the reviewer for his kind words and appreciate his comments regarding the bias toward Strand-seq. We believe it is a fair point and have restructured the text to be more balanced and more specific with respect to individual comparisons. Per Dr. Ebbert’s request, we have also posted a number of datasets and scripts onto Zenodo to facilitate reproducibility of presented results.

**Minor:**

1. The following statement likely needs to be expounded and qualified: "While various approaches have been developed over the years, the Strand-seq platform remains among the most sensitive".

ORIGINAL TEXT:

Even among existing high-quality long-read genome assemblies, large inversion polymorphisms are often missed or incorrectly represented [(Porubsky, Vollger, et al. 2022)](https://paperpile.com/c/Lh68gZ/YtLRj). While various approaches have been developed over the years to detect inversions, the Strand-seq platform remains among the most sensitive [(Porubsky, Höps, et al. 2022; Hanlon, Lansdorp, and Guryev 2022)](https://paperpile.com/c/Lh68gZ/fFxBv+QkwxO). Accurate detection of inversions is critical for understanding human variation and disease because recurrent inversions have been shown to associate with regions prone to rearrange and cause neurodevelopmental disease [(Osborne et al. 2001; Koolen et al. 2006; Cáceres et al. 2007; Zody et al. 2008; Cooper et al. 2011; Coe et al. 2014; Mohajeri et al. 2016)](https://paperpile.com/c/Lh68gZ/X7IVB+f5lqt+NbT5h+cpejj+bucHA+vJZWO+2u04j).

MODIFIED TEXT:

Even among existing high-quality long-read genome assemblies, large inversion polymorphisms are often missed or incorrectly represented [(Porubsky, Vollger, et al. 2022)](https://paperpile.com/c/Lh68gZ/YtLRj). While various approaches have been developed over the years to detect inversions (including mate-pair detection, optical mapping, Strand-seq, and long-read sequence detection), a combination of these methods has been shown to produce the best results. Long-read sequencing methods are particularly powerful for detecting smaller inversions (<10 kbp) that occur in relatively repeat-free regions of the genome. In contrast, the Strand-seq platform remains among the most sensitive for detection of large inversions (>10 kbp) that are flanked by large duplicated sequences [(Porubsky, Höps, et al. 2022; Hanlon, Lansdorp, and Guryev 2022)](https://paperpile.com/c/Lh68gZ/fFxBv+QkwxO) and affect the greatest number of base pairs per haploid genome. Accurate detection of inversions of this type is critical for understanding human variation and disease because recurrent inversions have been shown to associate with regions of genome instability and neurodevelopmental disease [(Osborne et al. 2001; Koolen et al. 2006; Cáceres et al. 2007; Zody et al. 2008; Cooper et al. 2011; Coe et al. 2014; Mohajeri et al. 2016)](https://paperpile.com/c/Lh68gZ/X7IVB+f5lqt+NbT5h+cpejj+bucHA+vJZWO+2u04j).

a. Potentially misleading and biased: While Strand-seq may be "among" the most sensitive, I feel like this statement is misleading and should be qualified. Specifically, it only mentions one of the approaches used in this paper (i.e., Strand-seq) and ignores the other methods used in this paper's primary results (i.e., standard long-read sequencing and optical DNA mapping)! Specifically, the second sentence in the results states the following: "…and combined it with both Bionano Genomics and assembly-based approaches to detect inversions". If (as scientists) we want to educate our readers without bias, we should be careful to educate them fully—especially in the introduction to the paper. If this paper were only using Strand-seq, I could understand why the introduction would only that Strand-seq is "among" the most sensitive, but the work clearly includes other approaches in the primary results, suggesting these other technologies added valuable information. As the authors

acknowledge, there is a clear conflict of interest related to Strand-seq. Please give credit where credit is due.

We agree with the reviewer that in a number of places bias towards Strand-seq might be misleading and we therefore modify our statements to emphasize that a combination of approaches produces the best results but also qualify precisely where Strand-seq outperforms the other methods (see modified introduction above).

b. Additionally, I believe sensitivity for each platform varies by structural variant size (e.g., Bionano often does better on the larger variants than standard long-read sequencing approaches, while long-read seq typically excels with smaller variants). I quickly read through one of the cited papers (Porubsky et al. 2022, Cell) to see if it supports the broad claim above. I was surprised to see that this paper makes an even broader statement, which may have been even more misleading. I could be wrong, but I couldn't find sufficient data in the Cell paper to support the broad claim. Specifically, the Cell paper acknowledges that standard long-read sequencing increases sensitivity for "events smaller than 100kbp", but then broadly states that "Bionano technology is least sensitive…", citing Figure 1D. Unless I misinterpreted Figure 1D in Porubsky et al. 2022 (Cell), I do not see any stratification by structural variant size to indicate that Bionano is "least sensitive" across

the entire spectrum of variant sizes. Nor did I see additional figures or stats supporting or clarifying this statement, but I may have overlooked it. I did not read the other cited papers, but it would helpful if the authors could either clarify/qualify throughout the paper, or persuade me that I am mistaken.

We agree with the reviewer that inversion detection sensitivity is platform dependent and each platform performs differently at different inversion size ranges. We revised our introduction to reflect this (see above).

We would like to note, however, that we have performed a direct comparison of various platforms, including Bionano genomics on the same DNA samples (Porubsky et al., 2022). Bionano is the least sensitive platform providing clear inversion signal for a subset of inversions within 100 kbp to 5 Mbp size range. Importantly, Bionano does not provide consistent genotypes across multiple samples with many missing genotypes. We compared both automated and manually curated callsets. The automated Bionano inversion callset was generated by Bionano representatives using Bionano Solve (v3.5), while the manually curated Bionano callset was performed by experts in this technology within the HGSVC group. We note, however, that improvements in the Bionano automated SV-calling pipeline (current Bionano Solve v3.7.1) may result in further improvements. Due to the fact that this comparison was made using GRCh38 inversion callset (automated Bionano calls available only for this reference) and also using an older version of Bionano SV-calling pipeline, we do not plan to add these results to the revised version of our manuscript.

**Figure: Inversion detection sensitivity between Bionano and Strand-seq callsets.**

**A-top**) Fraction of inversion calls from the final callset (GRCh38 balanced inversions calls across 30 shared samples) supported by Bionano (BNG) or Strand-seq (SSQ) automated or manually curated callsets based on required 50% reciprocal overlap across sample (x-axis) that share all callsets (TRUE, green - pass required 50% reciprocal overlap threshold). **B**) Fraction of large inversion calls (range 100 kbp – 5 Mbp) across shared samples from the final callset (see above) supported by Bionano (BNG) or Strand-seq (SSQ) automated or manually curated callsets based on required 50% reciprocal overlap. **C**) Sorted size distribution of final inversion callset (see above) where each dot represents percentage of reciprocal overlap with the Bionano (BNG) and Strand-seq (SSQ) automated and manual callset across all shared samples. Transparency of each dot reflects how many times given inversion was genotyped across all considered samples (n=30).

2. Same issue as above for the following statement in introduction: "We specifically focused on the analysis of Strand-seq data generated from previous…"

We agree with the reviewer that mentioning only Strand-seq in this context is incomplete as the final inversion callset, presented in this study, is based on the information from long-read-based assemblies as well as Bionano data for a subset of the genomes. We revised the text to reflect the use of multiple platforms instead of a hyperfocus on Strand-seq.

ORIGINAL TEXT:

The T2T-CHM13 assembly has been put forward as an improved human reference genome over the current incomplete GRCh38 and GRCh37 references. We sought to assess the potential advantage of detecting inversions on this new reference when compared to GRCh38 and whether it would significantly alter our understanding of the landscape and frequency of inversion polymorphism in the human genome. We specifically focused on the analysis of Strand-seq data generated previously from 41 human genomes (Porubsky, Höps, et al. 2022) and recalled inversions on the T2T-CHM13 reference. Our analysis uncovered orientation errors in the reference and identified novel inversion polymorphisms in previously inaccessible regions. We find that use of the new reference not only better represents minor and major inversion alleles but also provides new insights into enrichment within pericentromeric regions of the human genome. The work strongly suggests that this new reference should be adopted for human inversion discovery especially to discover new complex polymorphisms in more diverse human population samples—one of the goals of the Human Pangenome Reference Consortium (HPRC) [(Wang et al. 2022)](https://paperpile.com/c/Q0djDz/jnwCq).

MODIFIED TEXT:

The T2T-CHM13 assembly has been put forward as an improved human reference genome over the current incomplete GRCh38 and GRCh37 references. We sought to assess the potential advantage of detecting inversions on this new reference when compared to GRCh38 and whether it would significantly alter our understanding of the landscape and frequency of inversion polymorphism in the human genome. We, therefore, recalled inversions with respect to the T2T-CHM13 reference using data from multiple genomic platforms (Strand-seq, Bionano, and long-read assemblies) for 41 human genomes of diverse population origin (Porubsky, Höps, et al., 2022). Our analysis uncovered orientation errors in the reference and identified novel inversion polymorphisms in previously inaccessible regions. We find that use of the new reference not only better represents minor and major inversion alleles but also provides new insights into enrichment within pericentromeric regions of the human genome. The work strongly suggests that this new reference should be adopted for human inversion discovery especially to identify new complex polymorphisms in more diverse human population samples—one of the goals of the Human Pangenome Reference Consortium (HPRC) [(Wang et al. 2022)](https://paperpile.com/c/Q0djDz/jnwCq).

3. The following statement needs to be clarified either for me, or the readers (or both). i.e., why do you only focus on those variants? Why are you excluding the rest? "For the remainder of this study, we focus exclusively on analysis of 296 balanced inversions, 56 inverted duplications, and 21 complex events…" Those variants are at least as interesting as the balanced inversions.

Yes, we agree that these other classes of inversions are of interest; however, the inverted duplications and complex events pose additional challenges that are not currently resolvable using most methods, including Strand-seq. For example, regions classified as inverted duplications tell us nothing about the structure as they only point to an additional duplicated and inverted sequence that is often collapsed within assemblies. Similarly, complex events are difficult to genotype using Strand-seq due to the insufficient mappability of short Strand-seq reads and, thus, we can only tell that there might be a change in orientation in a given region but the full extent of complexity is difficult to resolve. We focused on balanced inversions primarily because they are genotypable and therefore can be comprehensively assessed in population sample sets to estimate accurate allele frequencies.

We now add a new paragraph to the **Supplemental Notes** describing our rationale and also added a sentence to the Results section to make clear the limitation of our presented inversion discovery and genotyping.

ORIGINAL TEXT:

For the remainder of this study, we focus exclusively on analysis of 296 balanced inversions on autosomes and chromosome X (and refer to these as inversions or inversion polymorphisms (Fig. 1A)

MODIFIED TEXT:

For the remainder of this study, we focus exclusively on analysis of 296 balanced inversions on autosomes and chromosome X and refer to these as inversions or inversion polymorphisms (Fig. 1A). We restrict our analysis to balanced inversions because they can be accurately and comprehensively genotyped using Strand-seq to establish meaningful population frequencies (**Supplemental Notes**).

**Supplemental Notes:**

**Rationale for focusing on balanced inversions:**

We focused on balanced inversions primarily because balanced inversions are more amenable to genotyping using Strand-seq data and, as such, we can estimate more accurate inversion population frequencies. In contrast, regions classified as inverted duplications are only telling us that a given region is duplicated and inverted in a given genome with respect to the reference. However, this is not conclusive as to where this duplicated region is positioned in the genome in question. Similarly, complex events are difficult to genotype using Strand-seq due to the insufficient mappability of short Strand-seq reads and, thus, we can only tell that there might be a change in orientation in a given region but the full extent of complexity is difficult to resolve.

4. Please forgive me if I'm misunderstanding, but the following statement in the results is a bit ambiguous. It may also be misleading depending on exactly which numbers are being compared. Please include the equation and percentage next to each stat for clarity: "Strand-seq detected the greatest proportion of inverted base pairs exclusively detecting 82 inversions with median size ~144kbp and corresponding to ~80Mbp of sequence."

a. As I understand, Strand-seq detected 82 of the 296 (82/296 = 28%) balanced inversions mentioned above? But those 82 account for ~80Mbp of the 82.8Mbp (~97%?) referenced above? Or is this 80Mbp of "all" inverted bases? Part of the reason this is unclear to me is that a preceding statement "clarifies" that the stats in that sentence are only "(considering balanced inversions only)". Incidentally, the results do not report the total number of *all* inverted bases. More summary statistics across all the variants would be extremely informative for the readers.

We thank the referee for identifying this confusing statement. As stated, it pointed to wrong summary statistics. We have modified the text accordingly.

ORIGINAL TEXT:

Consistent with earlier analyses [(Porubsky, Höps, et al. 2022)](https://paperpile.com/c/Q0djDz/Ex0e0), Strand-seq detected the greatest proportion of inverted base pairs exclusively detecting 82 inversions with median size ~144 kbp and corresponding to ~80 Mbp of sequence (Fig. S3).

MODIFIED TEXT:

Consistent with earlier analyses [(Porubsky, Höps, et al., 2022)](https://paperpile.com/c/Q0djDz/Ex0e0), Strand-seq detected the majority of large inversions (>10 kbp) exclusively detecting 82 inversions with median size ~144 kbp and corresponding to ~62.7 Mbp of sequence (Fig. S3A-D). Other technologies showed greater sensitivity for detecting smaller inversions (<10 kbp), especially less than 1 kbp. While more genes are inverted by the larger inversions, smaller ones are particularly important for detecting rare gene-disruptive events (Fig. S3E-F).

We modified Fig. S3 to include statistics on inverted bases detected exclusively by each technology and stratified by size as panel D (see below).

ORIGINAL:

**Figure S3: Inversion callset summary with respect to CHM13 reference.**

**A**) An upset plot showing the total number of inversions uniquely detected by each technology and those detected by two and more. **B**) A donut plot showing the number of inverted kilobases contributed separately by each technology to the final callset. **C**) A boxplot showing the size distribution of inversions uniquely detected by each technology and those detected by two and more (SHARED).

UPDATED:

**Figure S3: Inversion callset summary with respect to CHM13 reference.**

**A**) An upset plot showing the total number of inversions uniquely detected by each technology (‘PAV’ - phased-assembly; ‘BIONANO’ - Bionano Genomics, and ‘SSEQ’ - Strand-seq) and those detected by two and more (gray). **B**) A donut plot showing the number of megabases contributed separately by a single technology and those supported by more than one (gray). **C**) A size distribution of balanced inversions stratified by technology supporting each inverted site and those supported by more than one technology (gray - SHARED). **D**) Pie charts show proportions of balanced inversions (n=296), supported by each evaluated technology (see above) stratified per size. **E**) A barplot stratifying balanced inversions by size (x-axis) and for each size category showing the number of inversions (green), total number of overlapping protein-coding genes (orange), and number of inversion overlapping at least one protein-coding gene (purple). **F**) Fraction of inverted sites in each size category that overlap with at least one protein-coding gene.

We now also provide a size distribution for other inversion classes as Fig. S1B (see below); however, we still want to reserve reporting the total number of inverted bases only considering balanced inversions in order to avoid introducing biases caused by including complex inverted regions and inverted duplications whose exact copy number is difficult to ascertain from short-read data.

b. The reason this feels misleading is that it ignores that ~72% of the balanced inversions were presumably identified with the help of other approaches. Even if those 72% of events only account for ~3% of the nucleotides, they may be equally, or even more important. For example, maybe those 3% are directly affecting gene bodies or haplotypes of gene bodies, where perhaps the 97% of nucleotides are not. It would be great to see those stats, too. Of course, even if the 97% are not enriched for gene bodies, they may still be super important, but please provide more information!

We agree with the reviewer that statistics on inversion gene overlap stratified by size will be helpful. We added these statistics to **Fig. S3E,F** (see modified text above).

5. For figure 1A, the circles for "Inverted Allele Count" in the legend are not to-scale with what is presented in the figure itself, which makes the interpretation more ambiguous.

We thank the reviewer for pointing out this possible source of confusion. We adjusted the ‘Inverted allele count’ scale by adding extra breaks to the scale (1 and 10, and removing 80) to provide better size scaling for sites with low inversion allele counts (see updated figure below).

6. Would be helpful to indicate chromosome and region within figure 2B. I realize the text specifies, but it's not clear in the figure itself.

We now add the exact genomic coordinates of the plotted region to the upper left corner of Fig. 2B.

7. The methods state authors used LiftOver, but lift overs have a lot of inaccuracies. The authors state that all balanced inversions successfully lifted over. Please clarify how "success" is defined. Were these also manually curated?

This is of course a valid concern for most genome comparisons. In our case, this is mitigated by the fact that LiftOver is limited to T2T-CHM13 version 1.0 to version 1.1 reference comparisons where the only difference between the two is the addition of missing rDNA and improved polishing within telomeres. These are not regions where we have characterized inversions. Therefore, we expect lifted ranges to be accurate and largely concordant. We defined success as successful lift of ranges between v1.0 and v1.1 using liftOver tool and provided alignment chains. Nevertheless, we randomly selected 100 lifted inversion calls and checked sequence concordance between v1.0 and v1.1 of T2T-CHM13 references and found 100% concordance at the sequence level for all of them.

8. To properly educate the readers, when providing specific tool parameters in the methods, please indicate their meaning, purpose, and why the authors deviated from default.

We now add explanations of non-standard parameter settings to the relevant places in the methods section.

Occurrence #1:

ORIGINAL TEXT:

To extract FASTA sequence from a region of interest in T2T-CHM13 coordinates, we aligned available human assemblies from HPRC and HGSVC datasets to the T2T-CHM13 (v1.1) reference using minimap2 (version 2.24) with the following parameters: ‘-x asm20 --secondary=no -s 25000’. Next, we used rustybam (version 0.1.27) and its functionality called ‘liftover’ in order to subset alignments in PAF format to a region of interest.

MODIFIED TEXT:

To extract FASTA sequence from a region of interest in T2T-CHM13 coordinates, we aligned available human assemblies from HPRC and HGSVC datasets (Data availability) to the T2T-CHM13 (v1.1) reference using minimap2 (version 2.24) with the following parameters: ‘-x asm20 --secondary=no -s 25000’. Note an increased value was used for parameter -s in order filter out less contiguous alignments. Next, we used rustybam (version 0.1.27, 10.5281/zenodo.5875012) and its functionality called ‘liftover’ in order to subset alignments in PAF format to a region of interest (ROI).

Occurrence #2:

ORIGINAL TEXT:

To visualize Hi-C data, we first aligned short paired-end reads to the reference genome of interest (T2T-CHM13, v1.1). For this we used BWA (version 0.7.17) [(Li and Durbin 2010)](https://paperpile.com/c/Q0djDz/GmJLv) as follows: `bwa mem -5SP {input.ref} {input.pair1} {input.pair2}`.

MODIFIED TEXT:

To visualize Hi-C data, we first aligned short paired-end reads to the reference genome of interest (T2T-CHM13, v1.1). For this we used BWA (version 0.7.17) [(Li and Durbin 2010)](https://paperpile.com/c/Q0djDz/GmJLv) as follows: `bwa mem -5SP {input.ref} {input.pair1} {input.pair2}`. Note that set parameters -5SP are recommended to use only for the alignment of Hi-C data in order to obtain better alignments that are usually further apart than in standard mate-pair libraries.

Occurrence #3:

ORIGINAL TEXT:

To analyze each region of interest (ROI) in more detail, we aligned full genome assemblies (Data availability) to the T2T-CHM13 reference. We next used rustybam (version 0.1.27) (10.5281/zenodo.6342176) and its ‘liftover’ functionality in order to subset assembly-to-reference alignments to a certain ROI. We select only assembled contigs with a complete span of the ROI such that contig boundaries are no further than 100 kbp from left and right ROI coordinates. We reverse complement assembly FASTA sequence in case the first and last contig alignment of at least 50 kbp is in minus orientation with respect to the reference. This is done to synchronize orientation among all FASTA files. We use such FASTA files to visualize alignment directionality with respect to the reference (T2T-CHM13 v1.1). In the case of the 15q25.2 region, we also align each FASTA file to itself using minimap2 (version 2.24) with the following parameters: ‘-x asm10 -c --eqx -D -P --dual=yes -r10,50’. We record a relative orientation (reverse or direct) of such self-alignments within each haplotype and calculate fraction and the total length of these alignments. This information is then used as a proxy to predict if an intervening region (between flanking self-alignments) is predisposed to inversion or CNV.

MODIFIED TEXT:

We start by extracting FASTA sequence from a desired ROI as described in section ‘Extraction of FASTA sequence from a region of interest’. We select only assembled contigs with a complete span of the ROI such that contig boundaries are no further than 100 kbp from left and right ROI coordinates. We reverse complement assembly FASTA sequence in case the first and last contig alignments of at least 50 kbp are in minus orientation with respect to the reference. This is done to synchronize orientation among all FASTA files. We align such FASTA files from the ROI with respect to the reference (T2T-CHM13, v1.1) using minimap2 (version 2.24, parameters: -x asm20 --secondary=no -c --eqx -r 500,10k) in order to evaluate alignment directionality. In the case of the 15q25.2 region, we also align each FASTA file to itself using minimap2 (version 2.24, parameters: ‘-x asm10 -c --eqx -D -P --dual=yes -r 10,50’) to record a relative orientation (reverse or direct) of self-alignments within each FASTA file. Lastly, we select self-alignments that are at least 500 kbp apart and calculate the fraction and the total length of these self-alignments stratified by their relative orientation. We then use this information as a proxy to predict if an intervening region (between flanking self-alignments) is predisposed to an inversion or a CNV. Note that we have adjusted minimap2 parameter -r in order to decrease self-alignment redundancy as well as to obtain continuous alignments over short inverted regions (see minimap2 manual page for more details).

9. Unless I overlooked it, the authors do not provide their exact scripts/pipeline to reproduce their results. This should ideally also include a Singularity container, or related environment.

The majority of the presented analysis was performed using either open-access tools such as rustybam (https://github.com/mrvollger/rustybam) or already published R packages breakpointR, SaaRclust, or regioneR. Nevertheless, we now add a number of R scripts and preprocessed data to the Zenodo repository in order to help readers reproduce presented results.

ORIGINAL TEXT:

Availability of data and materials

HPRC assemblies used in this study have been reported in [(Liao et al. 2022)](https://paperpile.com/c/Q0djDz/RYC3c). HGSVC assemblies used in this study have been reported in [(Ebler et al. 2022)](https://paperpile.com/c/Q0djDz/sQoky). HPRC Strand-seq data: Data for eight samples (HG01123, HG01258, HG01358, HG01361, HG01891, HG02257, HG02486, HG02559) are available via ENA at accession number: PRJEB54100 [(Porubsky, Vollger, et al. 2022)](https://paperpile.com/c/Q0djDz/ziVPW). Additional Strand-seq data for samples HG01138 and HG01888, reported in this study, are available via ENA at accession numbers ERS13463611 and ERS13463612, respectively. Hi-C data for two samples (HG02011 and NA20847) presented in this study can be obtained at accession number: ERP123231 [(Ebert et al. 2021)](https://paperpile.com/c/Q0djDz/xjCr1).

MODIFIED TEXT:

Availability of data and materials

HPRC assemblies used in this study have been reported by [(Liao et al., 2022)](https://paperpile.com/c/Q0djDz/RYC3c). HGSVC assemblies used in this study have been reported by [(Ebler et al., 2022)](https://paperpile.com/c/Q0djDz/sQoky). Strand-seq data for eight samples (HG01123, HG01258, HG01358, HG01361, HG01891, HG02257, HG02486, and HG02559) reported by [(Porubsky, Vollger, et al., 2022)](https://paperpile.com/c/Q0djDz/ziVPW) including additional samples HG01138 and HG01888, used in this study, can be obtained via ENA accession number: PRJEB54100. Hi-C data for two samples (HG02011 and NA20847) presented in this study can be obtained at accession number: ERP123231 [(Ebert et al., 2021)](https://paperpile.com/c/Q0djDz/xjCr1). Additional data, including custom R functions and scripts used in this study, can be obtained at Zeno

**Second round of review**

**Reviewer 1:** I appreciate the authors addressing all of my concerns so well. It's a great paper.
